# Map the apps: a rapid review of digital approaches to support the engagement of older adults in strength and balance exercises

**DOI:** 10.1186/s12877-020-01880-6

**Published:** 2020-11-18

**Authors:** Lisa McGarrigle, Elisabeth Boulton, Chris Todd

**Affiliations:** 1 School of Health Sciences, Faculty of Biology, Medicine and Health, The University of Manchester, Jean McFarlane Building, University Place, Oxford Road, Manchester, M13 9PL UK; 2 Manchester Academic Health Science Centre, Manchester, M13 9NQ UK; 3 National Institute for Health Research (NIHR) Older People and Frailty Policy Research Unit, School of Health Sciences, Faculty of Biology, Medicine and Health, The University of Manchester, Manchester, M13 9PL UK; 4 Manchester University NHS Foundation Trust, Manchester, M13 9WL UK; 5 National Institute for Health Research, Applied Research Collaboration- Greater Manchester, Manchester, M13 9PL UK

**Keywords:** Apps, Websites, Strength, Balance, Exercise, Behaviour change techniques, Falls prevention, COVID-19

## Abstract

**Background:**

Exercise interventions, particularly those targeting strength and balance, are effective in preventing falls in older people. Activity levels are generally below recommended levels and reduce with age. There is concern that exercise levels may be further reduced in the context of the COVID-19 pandemic. Digital approaches may offer a means for older people to engage in strength and balance exercises independently in their own homes. The objective of this review was to identify and evaluate existing apps and websites to support independent engagement in strength and balance exercises by older people.

**Methods:**

We conducted a rapid review of apps and websites, following PRISMA guidelines. We searched for available apps in the Android and iOS app stores, and performed a database search (MEDLINE and EMBASE) for apps in development. We searched for websites using the Google search engine. Apps and websites were evaluated in terms of existing evidence for effectiveness, use of behaviour change techniques (BCTs), and quality.

**Results:**

We evaluated 13 apps and 24 websites on the basis of our selection criteria. Considering the evidence-base, quality and BCT scores, four apps and six websites are recommended for use by older people who wish to engage in exercise independently in their own homes. No apps or websites have been to RCT evaluation at the time of review.

**Conclusions:**

Apps and websites have the potential to provide a convenient, cost-effective, and accessible means for many older adults to engage in strength and balance training and reduce falls risk.

**Supplementary information:**

**Supplementary information** accompanies this paper at 10.1186/s12877-020-01880-6.

## Background

Worldwide, falls are the second leading cause of accidental death, with those aged over 65 experiencing the largest proportion of fatal falls [1]. Injury resulting from falls is associated with reduced physical functioning, loss of independence, and fear of future falls, which can in turn lead to reductions in physical activity and social engagement [2, 3]. A major causal factor in falls amongst older people is reduced muscle mass and strength resulting from metabolic changes and low levels of physical activity [4]. Exercise interventions, particularly those targeting strength and balance, can be effective in preventing falls in older people [5]. Recent UK Chief Medical Officers’ guidelines state that all adults aged 65 and over should aim to perform muscle-strengthening and balance exercises at least 2 days a week [6]. In the UK, rates of engagement in strength and balance activities are generally low, with only one in four women and one in three men over the age of 19 meeting the recommended guidelines, and the number meeting the recommendations decreases with age [7].

During the worldwide COVID-19 pandemic of 2020 there is concern that stay at home measures to reduce transmission of coronavirus will have adverse effects on older adults’ levels of activity with resultant deconditioning and increased falls risk. There is evidence from systematic reviews to suggest that exercise interventions delivered via apps and websites are successful in increasing physical activity levels in older people [8, 9], but similar evidence in relation to falls outcomes is lacking. There is also evidence that strength and balance training delivered via exergaming can improve strength and balance and reduce falls risk [10, 11], however, these kinds of interventions might not be immediately available to the general public. Given the risk the current pandemic restrictions pose to older people in relation to reduced activity, easily accessible and publicly available apps and websites that demonstrate strength and balance exercises via videos or images could act as potential substitutes in the absence of face-to-face exercise programmes. However, evidence for their effectiveness in falls prevention is unclear.

The objective of this review is to identify publicly available digital resources, in the form of apps and websites that can support older adults in performing strength and balance exercises independently, and to evaluate their quality, and evidence for effectiveness in improving strength and balance and/or preventing falls.

## Methods

We conducted a rapid review of apps and websites that support older adults in performing strength and balance exercises. The Prevention of Falls Network Europe (ProFaNE) [12] developed a taxonomy to classify different types of exercises. Strength exercises involve training the muscular system using free weights, body weight, or resistance, and may often involve an element of balance. Strengthening of the lower body is particularly important in falls prevention and examples of strength exercises may include heel and toe raises, knee bends, and sit to stand [13]. Exercises designed to improve balance involve the transfer of bodyweight from one part of the body to another and can include a wide variety of dynamic movements, such as heel and toe raises, squats, calf raises, and standing on one leg or tandem standing [12]. Although there is some evidence that “3D” exercises that involve constant movement, such as Tai Chi are also effective in falls prevention [5], for the purposes of this review we focused only on strength and balance exercises as outlined above. The review followed PRISMA guidelines. Rapid reviews allow for the synthesis of evidence relating to a specific query in a timely fashion [14, 15]. A protocol was registered on PROSPERO (CRD42020178582).

### Search strategy and selection criteria

The rapid review contained two search components: app search and website search. The app search was conducted between November 2019 and February 2020, and was re-run in May 2020 to identify any additional apps that had been recently developed. The website search was conducted in November 2019 and was re-run in May 2020 to ensure findings were up to date. One author (LM) conducted the searches and results were discussed with the other authors (EB, CT) to agree inclusion/exclusion.

#### App search

The first component involved: (a) a search of Android and iOS app stores for apps that met our inclusion criteria; (b) a database search for studies or protocols involving strength and balance exercise apps that are currently in development, but not yet publicly available. The app search was restricted to United Kingdom (UK) app stores. We did not exclude studies found during the database search on the basis of design (randomised controlled trials (RCTs), non-randomised studies or observational studies) in order to gain as full a view of the literature as possible. As this was a rapid review, apps and studies were restricted to the English language to ensure timely review and evidence evaluation.

The search was conducted using the following search strategy:

- Seven keyword searches were conducted in the Google Play android app store and the Apple App store. Health and Fitness app categories within each app store were searched using the following seven keywords/terms: falls prevention; balance exercise; balance training; strength exercise; strength training; strength and balance exercise; and strength and balance training. All terms were then combined with ‘older adults’, ‘elderly’ and ‘seniors’, giving a total of 28 searches in each app store.
- Database searches were conducted in MEDLINE (Ovid) and EMBASE (Ovid) for studies and protocols involving apps that met our inclusion criteria. Search terms are provided in Additional file 1.

#### Website search

The second component involved an internet search to identify websites that contained demonstrations (videos/images) of falls prevention exercises. The searches were conducted in the UK using the Google search engine. Keyword searches were performed using the following seven keywords/terms: fall prevention exercises; exercises to reduce falls; exercises to improve balance; balance training; strength training; strength and balance exercise infographics; and strength and balance exercise demonstration. These terms were searched in isolation and then in combination with ‘older adults’, ‘elderly’ and ‘seniors’, resulting in 28 searches. As research shows that internet users tend to explore only the first few hits from search engines, the first 20 results identified from each search were examined [16–18]. As such, results may not provide a fully comprehensive list of all available web-based falls prevention exercise demonstrations.

### Inclusion criteria

Apps and websites were included in the review if they:

- visually demonstrated strength and/or balance exercises (e.g. using videos or images); this was to ensure that the correct way to conduct the exercise was as clear as possible;
- were publicly available, or were in development with the intention of being made publicly available;
- could be used by older people independently, without supervision by a healthcare professional. As such, we will include apps and websites that target community-dwelling older people.
- apps were appropriate for use with older populations (i.e. were designed to be used by older people aged ≥50 years or had demonstrated use in older populations); the exercises demonstrated via websites were directly targeted at older people in relation to falls prevention;

### Exclusion criteria

We excluded apps and websites if they:

- provided only written descriptions of exercises;
- focused on improving specific disease conditions and falls prevention was a secondary outcome for the app intervention;
- targeted populations other than community-dwelling older people (e.g. patients in acute care facilities or care homes).
- focused on interventions that did not include strength and balance exercises (e.g. cognitive interventions for balance), or focused on general fitness;
- required specialist exercise equipment to work;
- for the website search, we did not consider material solely available on video sharing platforms (e.g. YouTube) or social media platforms (e.g. Facebook; Pinterest).

### Evidence evaluation

The quality of included apps and websites was evaluated as follows:

1. We determined the extent to which the exercises delivered via the app/website were based on evidence-based strength and balance programmes such as the Falls Management Exercise Programme (FaME) [19] or the Otago Exercise Programme (Otago) [20].
2. We determined whether the implementation of the exercise programme was theory-driven, e.g. drew on behaviour change techniques (BCTs) in order to change exercise-related behaviours. Two independent raters assessed this using the taxonomy of BCTs developed by Michie [21]. The taxonomy contains a list of 93 BCTs for which a score of “0” (absent) or “1” (present) is applied. Each app was awarded a total score ranging from 0 to 93, with higher scores indicating greater use of BCTs.

In addition, the quality of apps was assessed by at least two independent raters using a standardised tool for health apps, the Mobile Application Rating Scale (MARS) [22]. The scale contains 23 items assessing engagement, functionality, aesthetics, information quality, and subjective quality, and has demonstrated excellent internal consistency and inter-rater reliability. We also visited the app/developer websites and contacted the developers directly for information on any studies or evaluations that had been conducted in relation to the app. Where studies had been published, we planned to determine study quality using the evidence pyramid as a guide. As we did not anticipate finding any systematic reviews or evidence syntheses relating to the included apps, we considered RCTs as the highest quality evidence. Cohort studies and case-controlled studies were considered medium quality evidence. Non-randomised studies and studies with no control group were considered low quality evidence. Where possible, we planned to assess RCTs for bias using Cochrane’s Risk of Bias tool [23] and non-randomised studies for bias using the ROBINS-I [24]. We did not anticipate that effectiveness studies would have been conducted in relation to websites, so we merely checked the websites for any indications that evaluations had been conducted.

The quality of websites was assessed by two independent raters using two of the three criteria (credibility and senior friendliness) evaluated in Whitehead et al.’s [18] review of falls prevention websites. We did not grade based on the criterion ‘coverage of falls information’. This criterion was intended to grade the types of falls prevention information on websites, with information on strength and balance exercises considered Grade A information. As all websites were selected on the basis of providing information on strength and balance exercises, this criterion was excluded. Credibility and senior friendliness were assessed as follows:

1. Credibility: Assessed using the Health on the Net Code of Conduct for Medical and Health Websites (HONCode) [25], which consists of eight statements relating to factors such as authority and attribution of information, privacy, and transparency. A score of ‘1’ was awarded if the statement was satisfied, and ‘0’ if not. The first criterion, relating to medical advice, was not applicable to any of the websites, and so this item was not used. The score range for credibility was therefore 0–7.
2. Senior friendliness: Rated using a checklist developed by the National Institute on Aging. Forty requirements are listed across five categories: organising information (8 items); writing online text (11 items); designing readable online text (9 items); making information easy to find (9 items); and including other media (3 items). If the requirement was satisfied, a score of “1” was awarded, and a score of “0” if not, giving a score range of 0–40.

Scores for each category were summed to produce a total quality score ranging from 0 to 47. Percentage scores were calculated, and websites were categorised as poor, fair, good and excellent.

### Data analysis

Inter-rater reliability between two independent raters was assessed for BCT, credibility, and senior friendliness ratings using Krippendorff’s Alpha (Kalpha) [26], and all interpretations were based on mean rater scores. MARS ratings were conducted by multiple pairs of raters (*n* = 6), with only one rater remaining constant for all 13 apps. As such, there was substantial missing data across this small sample and inter-rater reliability analysis was considered inappropriate [27]. Differences in MARS scores, and the mean and standard deviation of the differences, were calculated between raters. Frequencies, means and standard deviations were calculated for the BCTs included in each app and website. Total scores on credibility and senior friendliness were converted into percentages and an overall website quality score was calculated. To ensure both categories were equally represented in the overall score, percentages for each individual category were calculated first, before a total average percentage was calculated. Scores less than 50% were interpreted as poor, scores between 50 and 62% as fair, scores between 63 and 75% as good, and scores greater than 75% were considered excellent [18]. Where possible, we planned to perform a process evaluation on studies found to have an effective intervention. We planned to summarise and tabulate five key elements outlined by the MRC [28] to identify common pathways. These included: description of the intervention; causal assumptions; implementation; mechanism of impact; and outcomes. Where there was sufficient homogeneity across studies evaluating the apps, we planned to conduct meta-analyses and derive forest plots to compare mean differences and 95% confidence intervals in rate of falls and number of fallers, as well as mean differences (95% CIs) across measures of strength, balance, cognitive and psychological factors associated with falling, between intervention and control groups.

## Results

**Fig. 1:**
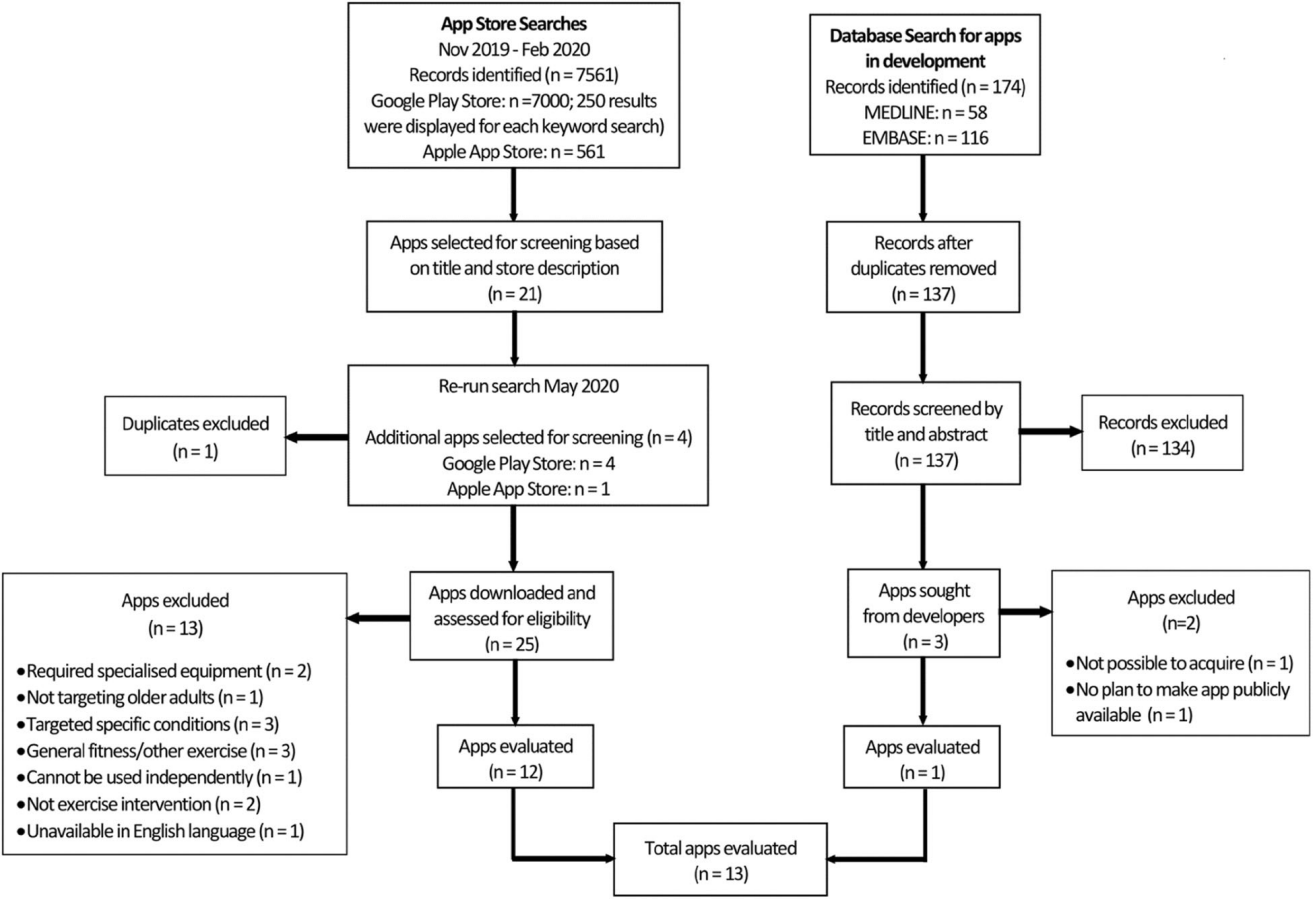
App search flow chart

**Fig. 2:**
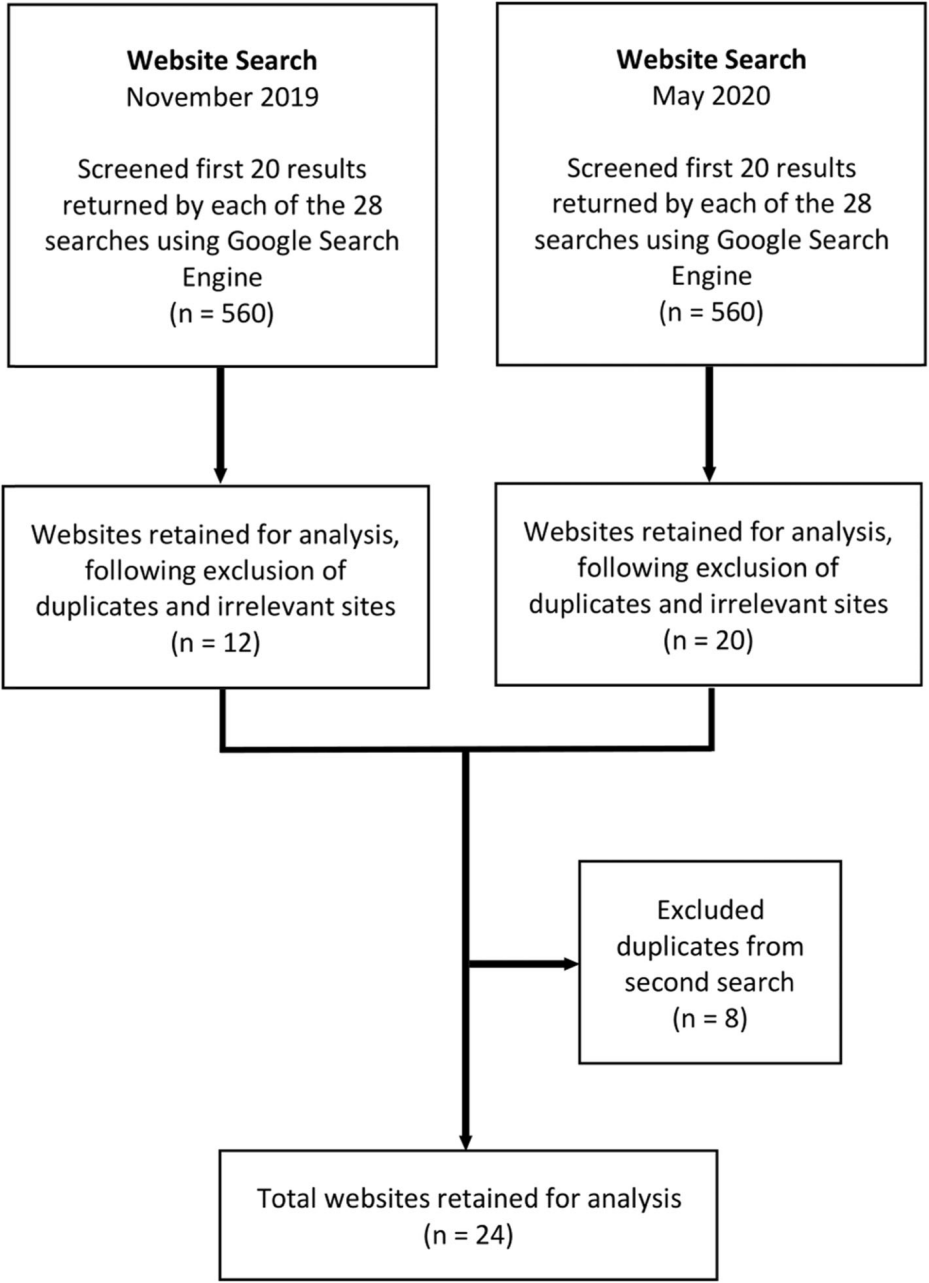
Website search flow chart

Flow diagrams of identification and retention of apps and websites are presented in Figs. 1 and 2. Overall, 28 apps were considered for inclusion. Reasons for exclusion following the app screening process are listed in Additional file 1. We identified 25 apps from the searches in Google Play and the Apple App Store. Of these, 12 fulfilled inclusion criteria (*LifeCurve; StopFalls; Otago Exercise Programme; Spiro100; Nymbl Balance Training; Moves4Me; Stannah Balance; Wysefit; Keep On Keep Up; Exercise Plan for Seniors; Hearty Seniors; and Senior Beginner Workout*). We identified three further apps from our database search for apps in development, one intervention study (*ActiveLifestyle*) [29] and two study protocols (*StandingTall* and *eLiFE*) [30, 31] involving app-based interventions. Out of these 15 apps, 11 were available for download in UK app stores. We contacted developers to gain access to the remaining four apps, and were granted access to two of them (*StandingTall; Nymbl Balance Training*). We were unable to gain access to the *ActiveLifestyle* app and the *eLiFE* app is not planned to be made publicly available following feasibility RCT testing [32]. Thus, these two apps were excluded from the review. A total of 13 apps were evaluated.

We identified 24 relevant websites for inclusion in the review. As there were no available published studies evaluating the effectiveness of the included apps or websites, we were unable to perform process evaluations, risk of bias assessments, or meta-analyses. Full narrative summaries of all apps and websites detailing the aim, target population, description, platform, and evidence evaluation are provided in Additional file 1.

### Characteristics of apps and websites

Ten of the 13 apps were commercially developed and three were developed by universities. Six were available on both Android and iOS platforms, four were iOS only, and three were Android only. Of the 12 apps currently available for download, eight were free to use, and four involved a subscription service. Most of the 24 websites originated from the USA (*n* = 11) or the UK (*n* = 6), with the remainder from Canada (*n* = 4), Australia (*n* = 1), Singapore (*n* = 1), and Europe (*n* = 1). Thirteen were provided by commercial organisations, six were provided by government, three were not-for profit, and two were academic. Detailed characteristics are provided in Additional file 1.

### App evaluation

Table 1 provides an evaluation summary of the 13 apps. On average, total MARS scores differed between raters by 0.57 points (SD = 0.38; difference range = 0.02–1.30). Inter-rater agreement between raters on BCT scores was strong (Kalpha = 0.89; 95% CI: 0.78, 0.97).

**Table 1:** App and website evaluation summary

App/website name	Evidence for exercise intervention *Yes/no/unclear*^a^	Evidence of effectiveness of app/website *Yes/no*	Mean BCT Score (SD) *Score out of 93 items*	Quality Rating (MARS score or website quality score)
*Apps*				
Exercise Plan for Seniors	Unclear	No	2.00 (0.00)	Good (3.60)
Hearty Seniors	Unclear	No	4.00 (0.00)	Acceptable (3.37)
Keep On Keep Up	Yes	No	6.50 (0.50)	Good (4.02)
LifeCurve	Yes	No	7.50 (0.50)	Acceptable/Good (3.50)
Moves4Me	Unclear	No	8.00 (0.00)	Acceptable (3.46)
Nymbl Balance^b^	Yes	No	6.50 (0.50)	Good (4.09)
Otago Exercise Programme	Yes	No	6.50 (0.50)	Good (3.57)
Senior Beginner Workout	No	No	4.00 (0.00)	Acceptable (3.45)
Spiro100	Unclear	No	2.00 (0.00)	Acceptable (3.38)
Standing Tall^c^	Yes	No	8.50 (0.50)	Good (3.78)
Stannah Balance	Yes	No	4.50 (0.50)	Acceptable/Good (3.50)
StopFalls	Yes	No	3.50 (0.50)	Acceptable (2.78)
Wysefit	Unclear	No	1.50 (1.50)	Good (3.83)
*Websites*				
ageuk.org.uk	Yes	No	3.00 (0.00)	Excellent (87%)
betterhealthwhileaging.net	Yes	No	4.00 (0.00)	Good (66%)
buffalorehab.com	Yes	No	4.50 (0.50)	Good (68%)
caregiverstress.com	Yes	No	3.00 (0.00)	Good (72%)
caringseniorservice.com	Yes	No	2.50 (0.50)	Fair (50%)
closingthegap.ca	No	No	2.00 (0.00)	Fair (58%)
csp.org.uk	Yes	No	5.00 (0.00)	Excellent (86%)
dailycaring.com	Yes	No	4.00 (0.00)	Good (71%)
eldergym.com	Yes	No	5.00 (0.00)	Fair (60%)
exerciseright.com.au	Yes	No	2.00 (0.00)	Excellent (81%)
fallsassistant.org.uk	Yes	No	7.00 (0.00)	Excellent (79%)
go4life.nia.nih.gov	Yes	No	7.00 (0.00)	Good (75%)
healthhub.sg	Yes	No	4.50 (0.50)	Good (73%)
healthline.com	Yes	No	4.00 (0.00)	Excellent (78%)
healthlinkbc.ca	Yes	No	4.00 (0.00)	Good (73%)
hopkinsmedicine.org	Yes	No	2.50 (0.50)	Good (68%)
mayoclinic.org	Yes	No	4.00 (0.00)	Excellent (85%)
melioguide.com	Yes	No	2.00 (1.00)	Good (64%)
nhs.uk/live-well	Yes	No	6.00 (0.00)	Excellent (81%)
nhsinform.scot	Yes	No	3.00 (1.00)	Excellent (83%)
pathsforall.org.uk	Yes	No	4.00 (0.00)	Good (72%)
preventfalls.ca	Yes	No	2.00 (1.00)	Good (69%)
profound.eu.com	Yes	No	5.00 (0.00)	Good (64%)
unitypoint.org	Yes	No	3.00 (0.00)	Fair (59%)

Overall quality for apps was assessed using MARS. Interpretation of MARS ratings is based on mean scores rounded to the nearest whole number (1 = Inadequate; 2 = Poor; 3 = Acceptable; 4 = Good; 5 = Excellent). Overall quality for websites was calculated as the mean of the total scores on credibility and senior friendliness and expressed as a percentage (poor = less than 50% score; fair = 50–62%; good = 63–75%; excellent = greater than 75%). BCT, MARS and website quality ratings reflect mean scores of two independent raters

^a^ ‘Yes’ = Promoted exercises are predominately evidence-based (e.g. feature in FaME or Otago programmes); ‘No’ = Few/none of the exercises feature in evidence-based programmes; ‘unclear’ = some of the exercises feature in evidence-based programmes

^b^ Only available for download in USA

^c^ In development and not yet publicly available

#### Use of evidence-based exercises

None of the apps have direct evidence supporting their effectiveness, and only one has recently been assessed using an RCT design, with results yet to be published (*StandingTall*). In seven apps, most of the promoted exercises featured in evidence-based exercise programmes, such as Otago and FaME (see Additional file 1 for information on the types of exercises included in each app). Five apps included at least some evidence-based exercises. One app (*Senior Beginner Workout*) did not contain any evidence-based exercises. Only one of the apps was explicitly based on an existing evidence-based exercise programme: the *Otago Exercise Programme* app (Otago).

#### Use of BCTs

**Fig. 3:**
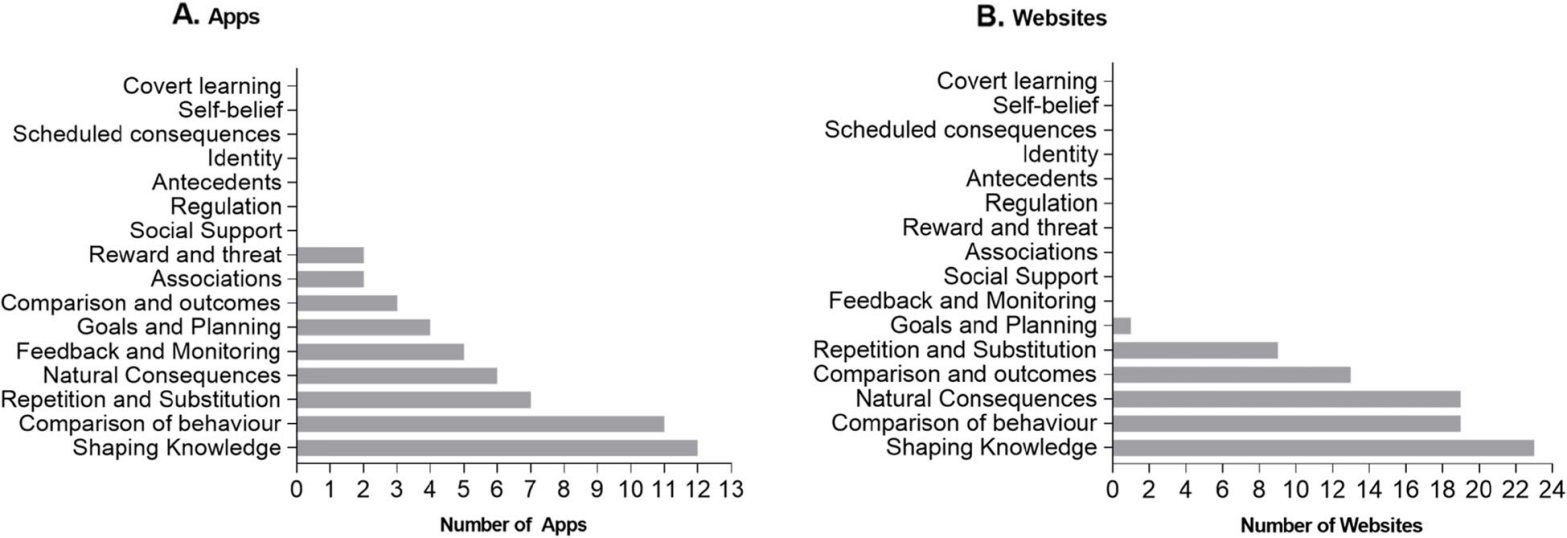
Frequency of BCTs by category across apps and websites. The figure illustrates the types of BCTs most frequently applied across apps and websites, based on categories provided in the 93-item BCT Taxonomy

The mean number of individual BCTs across all apps was five (SD = 2.30; range: 1.5–8.5). Figure 3 (panel a) illustrates the prevalence of BCTs across apps. Frequently included BCTs belonged to the following categories of the 93-item BCT taxonomy: shaping knowledge (12 out of 13 apps); and comparison of behaviour (11 out of 13 apps). Video-based apps (*Wysefit* and *Spiro100*) contained the fewest BCTs. Apps containing more than five individual BCTs (the top 50% in terms of the number of BCTs applied) included *StandingTall, Moves4Me, LifeCurve, Otago Exercise Programme, Nymbl Balance*, and *Keep On Keep Up*.

#### MARS quality ratings

The mean total MARS score was 3.56 out of 5 (SD = 0.32). Scores ranged from 2.78 (*StopFalls*) to 4.09 (*Nymbl Balance*). Most of the apps (8 out of 13) received a score of ‘good’, but two of these (*LifeCurve* and *Stannah Balance*) could be interpreted as ‘acceptable-good’ with scores falling almost exactly in between the two categories. Five of the apps were rated as ‘acceptable’ (Table 1).

### Website evaluation

Table 1 provides an evaluation summary for the 24 websites. Kalpha agreement was acceptable for credibility (0.77; 95% CI: 0.60, 0.89), and strong for senior friendliness (0.88; 95% CI: 0.83, 0.92) and BCT ratings (0.85; 95% CI: 0.73–0.95).

#### Use of evidence-based exercises

As expected, none of the websites have been subject to randomised trial design, or other evaluations of effectiveness. In 21 of the websites, most of the featured exercises also appear in evidence-based programmes. Two of the websites included at least some evidence-based exercises. Only one website (*closingthegap.ca*) did not appear to contain any evidence-based exercises. Three of the websites (*profound.eu.com, betterhealthwhileaging.net,* and *caringseniorservice.com*) contained video demonstrations of the Otago Exercise Programme, for which there is a strong evidence-base for effectiveness (see Additional file 1 for information on the types of exercises demonstrated by each website).

#### Use of BCTs

The mean number of individual BCTs across all websites was 3.88 (SD = 1.44; range: 2–7). Figure 3 (panel b) illustrates the prevalence of BCTs across websites. Frequently included BCTs belonged to the following categories of the 93-item BCT taxonomy: shaping knowledge (23 out of 24 websites); comparison of behaviour (19 out of 24); and natural consequences (19 out of 24). Websites containing more than 4.5 individual BCTs (the top 50% in terms of number of BCTs applied) included *buffalorehab.com, csp.org.uk, eldergym.com, fallsassistant.org.uk, go4life.nia.nih.gov, healthhub.sg, nhs.uk/live-well*, and *profound.eu.com*.

#### Quality of websites

Overall, the websites scored an average of 71.74%, indicating good quality. Eight were considered excellent, 12 were considered good, four were considered fair, and none of the websites were considered poor. The mean credibility score across all websites was 4.60 out of a possible 7 (65.77%) (SD = 1.15; range = 2–7), indicating that compliance with the HONCode standards for health-related websites was good. Only two websites stated that they were HONCode compliant, with *mayoclinic.org* meeting all seven standards, and *healthline.com* meeting six. Three of the websites (*caringseniorservice.com, eldergym.com*, and *unitypoint.org*), scored poorly on credibility (score < 50%) The mean senior friendliness score across all websites was 31.08 out of a possible 40 (77.71%) (SD = 4.13; range = 23–38), indicating that overall website senior friendliness was excellent. None of the websites scored lower than 20 (< 50%) on senior friendliness.

## Discussion

There is robust evidence for the effectiveness of community-based strength and balance exercise programmes in falls prevention, provided such programmes meet the minimum requirement of 50 contact hours over a 24 week period [5]. However, these types of structured programmes might not be feasible or accessible to many older adults living in the community, particularly during the COVID-19 pandemic where many older people are practising physical distancing. Digital approaches, such as apps and websites, offer a means of facilitating strength and balance exercises independently in the home. This review provides an overview and evidence summary of apps and websites that are publicly available or currently in development, to support older adult engagement in strength and balance exercises. We determined which of these apps appear most appropriate for home-based strength and balance exercise by identifying which apps scored well across our key evaluation criteria (Table 1). Of the seven apps that contained exercises with an evidence base in falls prevention, four also received a “good” MARS quality rating. These four apps (*StandingTall, Otago Exercise Programme, Nymbl Balance*, and *Keep On Keep Up*) also scored comparatively well on use of BCTs (i.e. scored in the top 50% in terms of the number of BCTs applied). Two of these apps are currently available for public download in the UK (*Otago Exercise Programme* and *Keep On Keep Up*) and could be recommended for use by older people who wish to engage in strength and balance exercises at home, particularly under the current pandemic conditions. *Nymbl Balance* may also be a useful tool, but it is currently only available for download in the USA. The *StandingTall* app is still under development and only available to services which have joined the Standing Tall-er Implementation Study (NIHR CPMS ID: 44434; IRAS ID 268954). Of note, none of these apps have published findings in relation to effectiveness in falls prevention, and it’s possible that although evidence-based exercises are promoted, online delivery of these programmes may lack key motivational and support elements present in face-to-face delivery.

We determined which websites may be most appropriate for independent strength and balance exercise by identifying which websites scored well across our key evaluation criteria (Table 1). Of the 23 websites that contained exercises with an evidence base in falls prevention, eight also received an “excellent” quality rating. Three of these websites also scored in the top 50% in terms of number of BCTs applied (*csp.org.uk, fallsassistant.org.uk*, and *nhs.uk/live-well*). These websites could be recommended to facilitate older adult engagement in strength and balance exercises in their own homes. Three additional websites (*profound.eu.com, betterhealthwhileaging.net,* and *caringseniorservice.com*) contained video demonstrations of the Otago exercise programme which has a strong evidence base. These websites may act as a substitute for face-to-face delivery for older people wishing to undertake evidence-based falls prevention exercise programmes in their own homes. The majority of the websites demonstrated some of the exercises that feature in falls prevention programmes with a strong evidence-base and as such, may prove useful for older adults wishing to engage in strength and balance training at home. However, as none of these websites have been evaluated in the context of an RCT or other study design, their effectiveness in falls prevention remains unknown. A key advantage of web-based videos and images is that they do not require a smartphone or tablet and are freely available to those with access to an internet-connected computer.

A strength of this review is the comprehensive search strategy and detailed quality appraisal of the featured apps and websites. Findings may also provide a useful summary for healthcare professionals looking for alternatives to face-to-face delivery of exercise programmes, particularly in the context of COVID-19. Exercise programmes are usually delivered by trained professionals during face-to-face classes or home visits, but this has largely been curtailed by COVID-19 lock down. This is a concern as the benefits of exercise are known to be lost rapidly once exercise is stopped [33]. Preliminary findings from this review were presented to the Department of Health and Social Care (DHSC) in the UK as part of a policy briefing on the digital delivery of strength and balance exercise training in response to increased home isolation and physical distancing [34]. Our report recommended that the apps and websites found to be of high quality following our evaluation could potentially be used as substitutes for face-to-face delivery. In addition, the recent Public Health England (PHE) prevention green paper [35] proposed to prioritise digital approaches in supporting the public to engage in strength and balance exercises regularly. Their aim is to provide digital products or services that are freely available to everyone, and support older people in particular, as well as those living with health conditions or people on low income. The apps and websites identified in this review could help to achieve PHE’s long-term aim, while also supporting older people to remain physically active during the COVID-19 pandemic. All of the recommended resources contain exercises used in existing face-to-face evidence-based programmes, although there is no evidence that these exercises are effective in falls prevention when delivered via a digital platform. Even so, the roll out of digital strength and balance exercises is preferable to older people not receiving any such interventions for the duration of the COVID-19 pandemic. The rigorous approach to app and website evaluation used in this review could be used by public health bodies to identify digital resources and build a “menu” for older people, carers, and healthcare professionals to select high quality, evidence-based digital tools that could facilitate exercising at home.

Even so, digital approaches may only act as a suitable alternative for generally healthy older people with experience using such technologies. Many older people may not have access to the internet, or internet-enabled mobile devices, particularly those from older age cohorts and poorer socio-economic groups, or those with declining visual acuity or cognitive function [36]. Furthermore, independent exercise may not be suitable for everyone, such as older adults with poor general health, frailty, or fear of falling [37]. This review was limited to searching for apps and websites in the UK, and as such, findings may not be generalisable internationally. In addition, as an indicator of quality, we assessed the extent to which apps and websites drew on BCTs using a simple count of the number of BCTs present according to a 93-item taxonomy [21]. Although several BCTs typically occur together in a given intervention and it is likely that the effect of a single BCT will be small, [38], the effect of specific BCT combinations in the context of app- and website-based exercise interventions on fall-related outcomes remains unclear. As such, conclusions about the behaviour change potential of these apps/websites due to BCT utilisation should be interpreted cautiously.

There is a need for a more thorough understanding of the effectiveness of digital approaches in falls prevention and this can be gained through RCTs of app- and web-based interventions. Websites may be more difficult to evaluate in a controlled manner as they are engaged with by users to varying degrees and under a range of conditions [39]. Despite these limitations, apps and websites have the potential to provide a convenient, cost-effective, and accessible means for many older adults to engage in strength and balance training independently and reduce falls risk.

## Conclusions

Apps and websites offer a means of facilitating strength and balance exercises independently in the home and may prove particularly useful as alternatives to face-to-face delivery of exercise programmes in the context of COVID-19. There are a number of high quality apps and websites currently available for use by older people who wish to engage in falls prevention exercises. Nevertheless, RCT evaluations of these kinds of approaches in falls prevention are needed.

## Supplementary Information

**Additional file 1.** Additional Information.

## Data Availability

Not applicable.

## Abbreviations

BCTs: Behaviour change techniques
HONCode: Health on the Net Code of Conduct for Medical Health Websites
MARS: Mobile Application Rating Scale
PHE: Public Health England
PRISMA: Preferred Reporting Items for Systematic Reviews and Meta-Analysis
RCT: Randomised controlled trial
