# Supplementary material for: Map the apps: a rapid review of digital approaches to support the engagement of older adults in strength and balance exercises
Source: BMC Geriatr. 2020 Nov 18;20:483. doi: 10.1186/s12877-020-01880-6 (PMC7673248; doi:10.1186/s12877-020-01880-6)
Supplement: Supplementary file 1 — Additional file 1. Additional Information. [file 12877_2020_1880_MOESM1_ESM.docx]

# Additional File 1: Additional Information

## MEDLINE (Ovid) and EMBASE (Ovid) Database Search Strategy

1. strength.mp. or Muscle Strength/

2. balance.mp. or Postural Balance/

3. fall*.mp. or Accidental Falls/

4. fall prevention.mp.

5. 1 or 2 or 3 or 4

6. Exercise/

7. exercise program*.mp.

8. exercise intervention.mp

9. 6 or 7 or 8

10. Smartphone/ or Mobile Applications/ or smart phone apps.mp.

11. Telemedicine/ or mobile technology.mp.

12. Computers, Handheld/

13. mobile health.mp

14. 10 or 11 or 12 or 13

15. 5 and 9 and 14

## Apps Excluded from the Review

Additional Table 1. Apps excluded from the review with reasons

|  | Apps excluded (with link to download or developer website) | Reason(s) for exclusion |
| --- | --- | --- |
|  | Sakra Steg (Android and iOS): Umeå University; <https://play.google.com/store/apps/details?id=se.umu.its.sakrasteg>  <https://apps.apple.com/us/app/s%C3%A4kra-steg/id1314353021> | Not available in the English language so could not be included in the evaluation |
|  | ActiveLifestyle (iOS): University of Trento, Italy & ETH Zürich; <https://itunes.apple.com/it/app/it4life-activelifestyle/id465295073?l=it&ls=1&mt=8> | Unable to download the app in the UK and the developers were unable to grant access |
|  | eLiFE (Android): PreventIT; <http://www.preventit.eu/> | Unable to gain access to the app through the developers |
|  | iFallCept (iOS): Legacy Star LLC;  <https://apps.apple.com/us/app/ifallcept/id1394521769>  <https://www.ifallcept.com/> | App did not meet inclusion criteria as it focuses on home adaptations in falls prevention |
|  | Fitivity Training (Android and iOS): Fitivity;  [https://play.google.com/store/apps/details?id=com.fitivity.stability_and_balance&hl=en_](https://play.google.com/store/apps/details?id=com.fitivity.stability_and_balance&hl=en_IE)  <https://apps.apple.com/us/app/flexibility-stretching/id738184221> | Did not meet inclusion criteria as not targeted at or tailored for older adults. |
|  | Get up and Go (iOS): New Digital Marketing Ltd.;  <https://apps.apple.com/app/get-up-and-go/id1470505716?ign-mpt=uo%3D5> | Did not meet inclusion criteria as the aim is to help prevent “PJ Paralysis” using exercises that can be done in a bed or chair. |
|  | Bobo Balance (iOS): BO & BO Ltd.;  <https://apps.apple.com/us/app/bobo-balance/id1266313426> | Did not meet inclusion criteria as required specialised equipment (balance board) |
|  | Bodyteamwork (Android): MFT Bodyteamwork;  [https://play.google.com/store/apps/details?id=air.at.shsoft.mft.coordi&hl=en_](https://play.google.com/store/apps/details?id=air.at.shsoft.mft.coordi&hl=en_IE) | Did not meet inclusion criteria as required specialised equipment (balance board) |
|  | Verticality Game (iOS): Inbal Gefen  <https://apps.apple.com/us/app/verticality-game/id971052483> | Did not meet inclusion criteria as targets individuals with a specific condition (Dysutonomia) |
|  | VOR neurobalance (Android and iOS): Paul Michael;  https://play.google.com/store/apps/details?id=com.xngamestudio.vorneurobalance&hl=en_  <https://apps.apple.com/us/app/vor-neuro-balance/id1200293029> | Did not meet inclusion criteria as intervention was not strength and balance (neuro intervention to coordinate visual and vestibular senses) |
|  | iPrescribe Exercise (iOS): DrFirst, Inc.;  <https://apps.apple.com/us/app/iprescribe-prescribe-anywhere/id1159038068> | Did not meet inclusion criteria as focused on general physical fitness rather than falls prevention |
|  | Parkinson’s Home Exercises (Android and iOS): European Foundation for Health and Exercise;  [https://play.google.com/store/apps/details?id=nl.efox.parkinsonss.en.phone&hl=en_](https://play.google.com/store/apps/details?id=nl.efox.parkinsonss.en.phone&hl=en_IE)  <https://apps.apple.com/us/app/parkinson-home-exercises/id473641730> | Did not meet inclusion criteria as targets individuals with a specific condition (Parkinson’s disease) |
|  | Vivifrail (Android): Vivifrail;  [https://play.google.com/store/apps/details?id=com.mikelizquierdo.vivifrail&hl=en_](https://play.google.com/store/apps/details?id=com.mikelizquierdo.vivifrail&hl=en_IE) | Did not meet inclusion criteria as cannot be used independently by user (healthcare professional led) |
|  | Fit and 50 PT (Android): BH app development Ltd.;  <https://play.google.com/store/apps/details?id=com.bhappdevelopment.laurencecodd&hl=en> | Did not meet inclusion criteria as focused on general physical fitness, rather than falls prevention. |
|  | Daily Senior Fitness Exercise (Android): EBMACS;  <https://play.google.com/store/apps/details?id=com.ebmacs.dailyseniorfitnessexcercise&hl=en> | Did not meet inclusion criteria as the aim of the exercise programme is to relieve pain. The majority of exercises are not related to strength and balance |

## Characteristics of Apps and Websites

Additional Table 2. Summary of app characteristics

| App Name | Developer | Platform | Cost  (May 2020) | Language |
| --- | --- | --- | --- | --- |
| *Exercise Plan for Seniors* | Samantha Roobol | iOS  Android | Free for some workouts. Subscription for specific exercise plans | English |
| *Hearty Seniors: Workouts for Seniors* | Hearty Seniors | Android | Free | English |
| *Keep On Keep Up* | University of Manchester;  Reason Digital | iOS (iPad only) | Free | English |
| *LifeCurve* | ADL Smartcare;  Newcastle University;  Later Life Training | Android  iOS  Amazon | Free | English |
| *Moves4Me* | Digital Health and Wellbeing Ltd. | Android  iOS | £8.49 for three months | English |
| *Nymbl Balance Training* | Nymbl Science, Inc. | Android  iOS | Free to user (cost covered by US health insurance) | English |
| *Otago Exercise Programme* | FOU I Sormland;  Later Life Training | Android | Free | English  Swedish |
| *Senior Beginner Workout* | App4Life | Android | Free | English |
| *Spiro100* | TV Free Media | Android  iOS | £9.49 per month / £92.99 per year | English |
| *Standing Tall* | NeuRA | iOS, but will be developed for Android and web-based platform | N/A (in development) | English |
| *Stannah Balance* | Stannah Stairlifts Ltd. | iOS | Free | English |
| *StopFalls* | Hertfordshire Care Providers Association | Android  iOS  Windows | Free | English |
| *Wysefit* | Wysefit Inc. | iOS | USD $8.99 per month | English |

Additional Table 3. Characteristics of websites

| Website | Country/ Region | Type of organisation | Website scope | Website Link | Specific strength and balance exercise content links |
| --- | --- | --- | --- | --- | --- |
| *Age UK* | UK | Registered charity | Information, advice, and services for older people | <https://www.ageuk.org.uk/> | <https://www.ageuk.org.uk/Documents/EN-GB/strength_and_balance_training_PDF.pdf?dtrk=true>. |
| *Better Health While Aging* | USA | Commercial | Information, advice, and educational resources on health various health issues impacting older people and caregivers | <https://betterhealthwhileaging.net/> | <https://betterhealthwhileaging.net/otago-exercises-fall-prevention-videos/> |
| *Buffalo Rehab* | USA | Commercial | Physical therapy services, advice, and information | <https://buffalorehab.com/> | [https://buffalorehab.com/blog/tips-to-improve-your-balance/#targetText=The%20next%20exercise%20you%20can,posture%20and%20maintain%20your%20balance.&targetText=If%20you%20are%20able%20to,can%20try%20single%20limb%20stance](https://buffalorehab.com/blog/tips-to-improve-your-balance/#targetText=The%20next%20exercise%20you%20can,posture%20and%20maintain%20your%20balance.&targetText=If%20you%20are%20able%20to,can%20try%20single%20limb%20stance.) |
| *Caregiver Stress* | USA | Commercial | General resources for caregivers of older adults | <https://www.caregiverstress.com/> | <https://www.caregiverstress.com/geriatric-professional-resources/share-clients/6-easy-at-home-exercises-to-reduce-senior-fall-risk/> |
| *Caring Senior Service* | USA | Commercial | Home care services for older adults | <https://www.caringseniorservice.com/> | <https://www.caringseniorservice.com/blog/great-fall-prevention-exercises-for-seniors> |
| *Chartered Society of Physiotherapy* | UK | Commercial | Information and resources relating to physiotherapy for professionals and patients | <https://www.csp.org.uk/> | <https://www.csp.org.uk/news/2017-09-27-csp-launches-video-demonstrate-six-simple-exercises-stop-falls>  <https://www.csp.org.uk/publications/get-and-go-guide-staying-steady-english-version>. |
| *Closing the Gap* | Canada | Commercial | Health services, information, and advice on a variety of health topics | [https://www.closingthegap.ca/](https://www.closingthegap.ca/blog/10-fall-prevention-exercises-seniors-can-do-while-sitting-in-a-chair/) | <https://www.closingthegap.ca/blog/10-fall-prevention-exercises-seniors-can-do-while-sitting-in-a-chair/> |
| *Daily Caring* | USA | Commercial | Information, advice, and resources for caregivers of older people | <https://dailycaring.com/> | <https://dailycaring.com/10-simple-fall-prevention-exercises-seniors-can-do-at-home-video/> |
| *Elder Gym* | USA | Commercial | Information, advice, and guidance on exercise for older people | <https://eldergym.com/> | <https://eldergym.com/elderly-balance/> |
| *Exercise Right* | Australia | Commercial | Exercise advice and guidance from professionals in exercise and sports science | <https://exerciseright.com.au/> | <https://exerciseright.com.au/keeping-simple-falls-prevention-exercises-home/> |
| *Falls Assistant* | UK | Government | Falls risk assessment and general advice and information on preventing falls | <https://fallsassistant.org.uk/> | <https://fallsassistant.org.uk/exercise-centre/> |
| *Health Hub* | Singapore | Government | General health information, advice, and e-services | <https://www.healthhub.sg/> | <https://www.healthhub.sg/programmes/71/healthy-ageing-exercise> |
| *Health Line* | USA | Commercial | General health information and resources | <https://www.healthline.com/> | <https://www.healthline.com/health/exercises-for-balance#exercise-for-seniors> |
| *Health Link BC* | Canada | Commercial | Medically approved information on a range of health topics and symptoms | <https://www.healthlinkbc.ca/> | <https://www.healthlinkbc.ca/health-topics/av2500> |
| *Johns Hopkins* | USA | Academic | Medical research, education, and clinical care | <https://www.hopkinsmedicine.org/> | <https://www.hopkinsmedicine.org/health/wellness-and-prevention/fall-prevention-exercises> |
| *Mayo Clinic* | USA | Non-profit | Medical research, education, and clinical care | <https://www.mayoclinic.org/> | <https://www.mayoclinic.org/healthy-lifestyle/fitness/multimedia/balance-exercises/sls-20076853?s=1> |
| *Melio Guide* | Canada | Commercial | Physiotherapy services, information, and resources | <https://melioguide.com/> | <https://melioguide.com/health-guides/balance-exercises-for-seniors/> |
| *National Institute on Aging* | USA | Government | General information on health topics of interest to older people | [https://www.nia.nih.gov](https://www.nia.nih.gov/health/exercise-physical-activity) | <https://www.nia.nih.gov/health/four-types-exercise-can-improve-your-health-and-physical-ability> |
| *NHS Inform* | UK | Government | General health information | <https://www.nhsinform.scot/> | <https://www.nhsinform.scot/healthy-living/preventing-falls/keeping-well/strength-and-balance-exercises> |
| *NHS Live Well* | UK | Government | General health advice and resources | <https://www.nhs.uk/live-well/> | <https://www.nhs.uk/live-well/exercise/strength-exercises/>  <https://www.nhs.uk/live-well/exercise/balance-exercises/>  <https://www.nhs.uk/live-well/exercise/strength-and-flex-exercise-plan/> |
| *Paths for All* | UK | Registered charity | Information and resources to encourage increased walking and physical activity | <https://www.pathsforall.org.uk/> | <https://www.pathsforall.org.uk/strength-and-balance-exercises> |
| *Prevent Falls* | Canada | Government | Information on falls prevention | <http://preventfalls.ca/> | <https://preventfalls.ca/older-adults/exercise/home-exercise/> |
| *ProFouND* | Europe | Academic | Information and resources on best practice in falls prevention | <https://profound.eu.com/> | <http://profound.eu.com/category/videos/page/2/>  <http://farseeingresearch.eu/> |
| *Unity Point* | USA | Commercial | Health services and information on general health topics | [https://www.unitypoint.org/](https://www.unitypoint.org/livewell/article.aspx?id=91b1a159-d94a-4ba2-b64e-81236a3d538a) | <https://www.unitypoint.org/livewell/article.aspx?id=91b1a159-d94a-4ba2-b64e-81236a3d538a> |

## Narrative Summaries of Apps and Websites

### Apps

1. *Exercise Plan for Seniors – Samantha Roobol*

Intention/Aim: Exercise Plan for Seniors is an exercise app that aims to help older adults establish an exercise routine and incorporate 150 minutes of moderate endurance activity into each week.

Target Population: Older adults

Description: The app provides four free workouts: Improve Strength and Balance; Yoga Poses; Dumbell Workout; and Stretching Exercises. Strength and balance workouts include warm up exercises; heel toe walking; chair squats; lunges; one leg balance; clock reach; and bicycle crunches. Each exercise is described in text and accompanying images. Each exercise is timed and the user can select the duration of the rest period in between each exercise. The total minutes spent exercising, total calories burned, and number of workouts completed is displayed on the home screen for the user to monitor. The app also provides six exercise programmes that are available at a cost: Stretching Exercises; Improve Balance; Yoga Poses; Strength Training – Bodyweight; Strength Training – Dumbbells; and The 60-Day Challenge.

Platform: iOS and Android

Evidence Evaluation: The Exercise Plan for Seniors App includes some exercises that also feature in evidence-based intervention programmes, such as FaME and Otago. The effectiveness of the app exercise programme in improving strength and balance and preventing falls is unknown. Based on two independent ratings, there were two to three BCTs embedded in the app relating to shaping knowledge, comparison of behaviour, and repetition and substitution. There is no evidence available regarding the effectiveness of the app. The overall MARS score was 3.6 out of 5 (good). Functionality was the highest scoring domain (3.9), followed by Aesthetics (3.7), Information Quality (3.5) and Engagement (3.4).

Links:

<https://apps.apple.com/za/app/exercise-plan-for-seniors/id1499804902#?platform=iphone>

<https://play.google.com/store/apps/details?id=senior.fitness.exercises.elderly&hl=da>

1. *Hearty Seniors: Workouts for Seniors – Hearty Seniors*

Intention/Aim: The Hearty Seniors app was created by physical therapists with the aim of improving mobility and strength in older adults.

Target Population: Older adults

Description: The app provides exercise programmes for both strength and mobility. Users are instructed to perform the exercises 2-3 times each week for a 6-week period. There are two levels to the strength training programme. The first contains eleven exercises including marching, shoulder circles, step back reach, dynamic chest stretch, squats, lunges and trunk rotation. The second contains eleven exercises including lunges, one leg stand, shoulder press, squats, push up, and chair planks. The mobility programme contains eleven exercises including heel toe walking, trunk rotation, glute bridge, dynamic knee to chest stretch. Exercises are described in text and accompanying videos.

Platform: Android

Evidence Evaluation: Some of the exercises included in the exercise programmes also feature in evidence-based intervention programmes, such as FaME and Otago. The effectiveness of the app exercise programme in improving strength and balance and preventing falls is unknown. Based on two independent ratings, there were four BCTs embedded in the app relating to the subscales of shaping knowledge, natural consequences, comparison of behaviour, and comparison and outcomes, which may facilitate behaviour change and promote adherence. There is no evidence available regarding the effectiveness of the app. The overall MARS score was 3.4 out of 5 (acceptable). Functionality was the highest scoring domain (4.3), followed by Information Quality (3.5), Aesthetics (3.2), and Engagement (2.6).

Links:

<https://play.google.com/store/apps/details?id=com.hearty.seniors.workout>

1. *Keep On Keep Up (KOKU) – University of Manchester and Reason Digital*

Intention/Aim: Keep On Keep Up (KOKU) is an NHS-approved tablet based app that aims to assist older adults to improve strength, balance, and optimise healthy ageing.

Target Population: Older adults

Description: The app sets three daily exercises that start as light seated workouts and increase to more challenging standing routines as the user progresses through the programmes. They are designed to help improve strength, balance and independence. The app allows the user to select their general ability level from a predefined list. A walkthrough demonstrates the apps functions. On completion of each exercise the user enters how many repetitions they did in order to track progress. Exercises include sit to stand, heel raises, leg lifts, lunges, heel toe walking, and backwards walking. KOKU is based on health behaviour change theory and gamification to increase adherence to strength and balance exercises.

Platform: iOS (currently iPad and UK download only)

Evidence Evaluation: The KOKU exercise programme includes exercises that also feature in evidence-based intervention programmes, such as FaME and Otago. As KOKU is still in development, its effectiveness in improving strength and balance and preventing falls is currently unknown. Research to date has included initial user-centred iterative development and testing with 58 older adults and 11 multi-disciplinary healthcare professionals (11 focus groups), for qualitative feedback [1,2]. A mixed methods, multi-centre, pre-post feasibility study was conducted in 2019 and findings are currently in preparation for publication [3]. Personal communication with the authors indicated that although the study was not powered (n=30), a trend towards an improvement in strength and balance scores in older adults residing in assisted living facilities (aged between 66 and 84 years) was apparent. KOKU is based on behaviour change theory and, based in two independent ratings, there were between 6 and 7 BCTs embedded in the app to encourage behaviour change and promote adherence relating to goals and planning, feedback and monitoring, shaping knowledge, natural consequences, comparison of behaviour, repetition and substitution, and reward and threat. The overall MARS score was 4.0 indicating good overall quality. The highest scoring domain was Functionality (4.4), followed by Aesthetics (4.3), Engagement (3.7), and Information Quality (3.7).

Links: <https://kokuhealth.com/>

<https://apps.apple.com/gb/app/keep-on-keep-up/id1155051089>

1. *LifeCurve – ADL Smartcare, Newcastle University and Later Life Training*

Intention/Aim: The ADL LifeCurve™ was developed by ADL Research and Newcastle University's Institute for Ageing as a mapping tool for functional decline. The tool can be used to determine which interventions are most effective and when it is most appropriate to implement an intervention.

Target Population: Older adults

Description: The LifeCurve™ App's fitness and strength exercises were created in collaboration with Later Life Training™. The app is self-managed and can tailor an individual’s exercise and advice to their ability level. The overall aim of the app is to help older adults remain independent for longer and reduce the cost of long-term care. The app can also be used by healthcare professionals to assess or monitor functional performance. Users select the exercises they would like to complete from a “task” menu that either directs them to in-app exercises or directs them to external links with exercise demonstrations tailored to ability level (NHS approved website “fallsassistant.org.uk”). Exercises related to strength and balance include heel raises, toe raises, heel toe stand, heel toe walking, flamingo swings, toe walk, heel walk, tandem walk, side stepping, sit to stand, knee lifts/marching, and knee bends. Exercises are described in text and demonstrated using, pictures, animations or videos.

Evidence Evaluation: The fitness and strength exercises were created in collaboration with Later Life Training ([www.laterlifetraining.co.uk](http://www.laterlifetraining.co.uk)) who use evidence-based exercises in their falls prevention programmes. The specific exercise programme is of unknown effectiveness. There is no evidence available regarding the effectiveness of the app. Based on two independent ratings there were between 7 and 8 BCTs embedded in the app in the form of goals and planning, feedback and monitoring, shaping knowledge, natural consequences, comparison of behaviour, associations, and repetition and substitution, which may facilitate behaviour change and promote adherence. The overall MARS score was 3.5 out of 5 (good). Aesthetics was the highest scoring domain (4.3), followed by Information Quality (3.5), Functionality (3.3), and Engagement (2.9).

Links: <https://www.adlsmartcare.com/LifeCurveApp>

<https://play.google.com/store/apps/details?id=so.appt.adl>

<https://apps.apple.com/us/app/lifecurve/id1432726960>

1. *Moves4Me - Digital Health and Wellbeing Ltd.*

Intention/Aim: Moves4Me aims to support older adults to stay physically stronger for longer by improving access to resistance exercise training.

Target Population: Older adults aged over 50

Description: The app is specifically designed to promote strength and balance, and prevent falls in older adults. Users undergo an initial assessment that includes the FES-I [4] and the Modified 30 Second Chair Stand [5] to determine the most appropriate level of exercises. Exercise programmes use instructional videos to demonstrate each exercise and the challenge level increases as the user improves their strength and balance. Exercises include sit-to-stand, knee raises, leg raises, assisted squats, wall press, and marching in place. Users are also able to track their improvements through activity-based reports and assessments. Users receive stars for regular exercise completion as a means of encouraging adherence. There is also the option to meet other users in a Moves4Me forum.

Platform: iOS and Android

Language: English

Cost: £8.49 for three months membership (sign up via their website). App is free to download and use once you have created an account with them. Two-day free trial available.

Evidence Evaluation: The app uses evidence-based assessments and some of the exercises included in the app are also included in interventions for which there is an evidence base of effectiveness (e.g. Otago, FaME). It is unclear if the Moves4Me exercise programme is based on an existing evidence-based intervention. The specific exercise programme presented is of unknown effectiveness. A poster was presented at a local conference outlining plans for a pilot study. Based on two independent ratings there are 8 BCTs embedded in the app to encourage behaviour change and promote adherence, relating to goals and planning, feedback and monitoring, social support, shaping knowledge, comparison of behaviour, comparison and outcomes, repetition and substitution, reward and threat. The overall MARS score was 3.5 (good). The highest scoring domain was Functionality (3.8), Information Quality (3.6), followed by and Engagement (3.5), and Aesthetics (3.0).

Links:

<https://apps.apple.com/us/app/moves4me/id1410248132>

<https://play.google.com/store/apps/details?id=com.moves4me.moves4meand&hl=en_GB>

<https://moves4me.com/>

1. *Nymbl Balance Training – Nymbl Science, Inc.*

Intention/Aim: The aim of the app is to use digital technology to deliver evidence-based, sustained balance interventions to older adults in order to prevent falls.

Target Population: Older adults

Description: Nymbl uses a “patent-pending dual-task approach” to balance improvement that includes both physical and cognitive challenges. The app demonstrates exercises that progress in challenge as the user’s balance improves. The recommended dose is 10 minutes of exercise three times a week. On first use of the app the user completes a pre-training STEADI assessment [6] in order to tailor the exercise programme to suit their ability level. Exercises increase in difficulty as the user progresses, and include exercises such as tandem stand, standing weight shift, knee lifts, side leg lifts, dynamic stepping, and single leg balance. These exercises are performed alone, and in combination with cognitive tasks. The Nymbl app can be used in a clinical setting or at home and all interactions can be monitored remotely. At present, there is no direct path between the app and consumers. In the USA, the app is provided to individuals via organisations such as homecare or physical therapists. Although Nymbl can be found in UK app stores, personal communication with the developers suggests they have no current plans to roll out in the UK.

Platform: iOS and Android

Language: English

Cost: Nymbl is covered under US health insurance and no costs are passed on to the user.

Evidence Evaluation: On the Nymbl website they state that a pre-post study conducted by the MSk lab at Imperial College London and presented recently at the 8th World Congress of Biomechanics conference, showed that Nymbl use over 21 days significantly improved stability (postural sway; sway velocity; medio-lateral sway; anterior-posterior sway). The website also reports the findings from pre-post studies they conducted in primary care and senior-living community settings that showed that 30 days of balance training improved balance in independent older adults. However, these findings have not been published and critical appraisal of the research overall is thus not possible. Nonetheless a non-randomised pre-post design is weak. Many of the exercises included in the Nymbl balance programme have featured in evidence-based falls programmes such as Otago and FaME. It is unclear if the Nymbl exercise programme is based on an existing evidence-based falls intervention programme. Based on two independent ratings, Nymbl has employed between 6 and 7 BCTs to encourage behaviour change and adherence, including feedback and monitoring, shaping knowledge, natural consequences, comparison of behaviour, associations, repetition and substitution, . The overall MARS score was 4.1 (good). The highest scoring domain was Functionality (5.00), followed by Engagement (4.0), Information Quality (3.7), and Aesthetics (3.7).

Links:

<https://apps.apple.com/us/app/nymbl-balance-training/id1155575069>

<https://play.google.com/store/apps/details?id=com.b4g.nymbl&hl=en_IE>

<https://nymblscience.com/the-nymbl-training-app/>

1. *Otago Exercise Programme – FOU i Sormland and Later Life Training*

Intention/Aim: The Otago Exercise app was developed by FOU i Sormland, a social services R&D group in Sormland, Sweden. Later Life Training worked with the app developers to ensure its translation into the English language format was accurate. The aim of the app is to support those who want prompts and advice on home exercise, to support group delivery of the Otago programme, or as a home exercise only app.

Target Population: Adults over the age of 65.

Description: The programme provides structured exercises for leg strength and dynamic balance exercises. The exercises take about 30 minutes and are recommended to be performed three times a week. The level of difficulty can be adjusted and it is recommended that ankle weights are used with some of the exercises.

Platform: Android

Cost: Free

Language: English; Swedish

Evidence Evaluation: The app is based on the Otago Exercise Programme, an intervention for which there is strong evidence for effectiveness. Based on two independent ratings, the app includes between 6 and 7 BCTs to encourage behaviour change and promote adherence to the programme. These include feedback and monitoring, shaping knowledge, natural consequences, comparison of behaviour, repetition and substitution, and comparison and outcomes. There is no evidence for the effectiveness of the app in relation to improved strength and balance and falls prevention. The overall MARS score was 3.6 (good). The highest scoring domain was Information Quality (4.0), followed by Functionality (3.9), Engagement (3.2) and Aesthetics (3.2).

Links: <https://play.google.com/store/apps/details?id=se.delphie.otago.classic>

<http://www.m-otago.com/>

<https://www.laterlifetraining.co.uk/support-home-exercise-with-the-otago-app/>

1. *Senior Beginner Workout – App4Life Developers*

Intention/Aim: The Senior Beginner Workout app aims to keep older adults healthy for longer through engagement in physical activity.

Target Population: Older adults aged 50+

Description: The app provides a 20-minutes workout specifically designed for adults aged over 50 years. The exercises require access to a sturdy chair and two equally weighted objects to use as dumbbells (e.g. soup cans or water bottles). The workout features warm-up, strength, flexibility, and balance exercises. Exercises can be modified to meet different needs. Exercises include squat curl knee lifts, shoulder overhead press, glute bridge exercise, and mid-back extensions, and video descriptions are provided for each.

Platform: Android

Evidence Evaluation: The exercises included in the app target strength and balance, however none of the exercises appear to be based on those featured in evidence-based intervention programmes, such as FaME and Otago. The effectiveness of the app exercise programme in improving strength and balance and preventing falls is unknown. Based on two independent ratings there are 2 BCTs embedded in the app, including shaping knowledge, natural consequences, comparison of behaviour, and comparison and outcomes, which may facilitate behaviour change and promote adherence. There is no evidence available regarding the effectiveness of the app. The overall MARS score was 3.5 out of 5 (good). Functionality was the highest scoring domain (4.3), followed by Information Quality (3.5), Aesthetics (3.5), and Engagement (2.5).

Links:

<https://play.google.com/store/apps/details?id=com.andromo.dev516135.app1015140>

1. *Spiro100 – TV Free Media*

Intention/Aim: The app aims to keep older people active and independent by providing over 100 fitness and wellness class videos that can be performed independently in the home.

Target Population: Older adults and caregivers of people with Alzheimer’s or dementia

Description: The app lists falls prevention as one of its specialities. It provides video demonstrations of strength and balance exercises that can be viewed via smartphone, tables, or smart TVs’ (Apple TV; Chromecast). “Fall-proof Masterclasses” are also provided. These classes include videos of exercise classes demonstrating exercises such as seated leg movements, altered bases of support, standing weight shifts, marching in place, and side leg lifts. There is also a class combining brain games with balance exercises. Some of the classes also have the option to include resistance bands to build strength. Activity level ranges from fully seated to advanced. Over 25 of the classes provided are stated to be appropriate for use with people with memory difficulties. A range of other classes are also provided (dance, toga, Tai Chi, cardio, posture). The app allows the user to search for classes using key words (e.g. balance) and add them to a library of favourite classes.

Platform: iOS and Android

Cost: £9.49 per month after a 7-day free trial. £92.99 for an annual subscription.

Language: English

Evidence Evaluation: Some of the exercise videos in this app feature exercises that have also been included in interventions for which there is an evidence base of effectiveness, such as Otago. It is unclear if the exercise programme is directly based on an evidence-based intervention. There is no evidence regarding the effectiveness of the app in improving strength and balance or preventing falls. Based on two independent ratings there were 2 BCTs embedded in the app, including shaping knowledge and comparison of behaviour. The overall MARS score was 3.4, indicating acceptable. The highest scoring domain was Functionality (4.1), followed by Aesthetics (3.2), Engagement (3.2) and Information Quality (3.0).

Links:

<https://apps.apple.com/us/app/spiro100/id1361393341>

<https://play.google.com/store/apps/details?id=com.spiro100&hl=en_US>

<https://spiro100.com/>

<https://spiro100.spirofit.com/> (some exercise videos)

1. *Stannah Balance (UK)- Stannah Stairlifts Ltd.*

Intention/Aim: Developed by the company behind Stannah Stairlifts, this app is designed to help older people monitor and improve their balance, reduce falls risk, and reduce fear of falling. It may also be useful to healthcare professionals who wish to monitor their patient’s balance. The app was developed in conjunction with leading Occupational Therapy professionals, the Royal College of Art, and potential users.

Target Population: Older adults

Description: According to the website the smart device, with app running, is placed in the user’s pocket to measure balance and provides audio instructions for a variety of simple movements across three difficulty levels: level 1- semi-tandem, level 2-tandem, and level 3-uni-pedal stands. There are a variety of stages of difficulty and movements become more challenging as the user progresses. However, it seems that the app as currently available (March 2020) has been pared back before release and now does not appear to measure any balance metrics, as was originally intended. It simply guides the user through balance exercises but does not provide feedback. As such it is unclear why the phone must be placed in the pocket.

Platform: iOS

Cost: Free

Language: English

Evidence Evaluation: The exercises included in the app are also included in interventions for which there is an evidence base of effectiveness, such as Otago. The specific exercise programme presented is of unknown effectiveness. Based on two independent ratings there are between 4 and 5 BCTs embedded in the app to encourage behaviour change and promote adherence, including goals and planning, shaping knowledge, natural consequences, comparison of behaviour, repetition and substitution, and reward and threat. The overall MARS score was 3.5 (good). The highest scoring domain was Aesthetics (4.7) followed by Information Quality (3.3), Engagement (3.2) and Functionality (2.9).

Links:

<https://apps.apple.com/us/app/stannah-balance/id1291480437>

<https://www.stannah.co.uk/stannah-news/stannah-stairlifts-launches-innovative-balance-app/>

1. *StopFalls - Hertfordshire Care Providers Association*

Intention/Aim: The StopFalls app was launched as part of Hertfordshire County Council’s StopFalls Campaign that aims to reduce falls through exercises, risk assessments and other prevention techniques. The app includes descriptions of exercises, information on falls risk factors, and risk assessments. The assessments can be shared with healthcare professionals for expert advice.

Target Population: Older adults living in the community, and care staff.

Description: The app contains information on how to manage falls risk, and describes and demonstrates exercises that can improve strength and balance. Users are encouraged to complete a risk assessment, the FRAT [7], prior to beginning the exercises. Links to other assessments that may be of interest to care providers are the FES-I [4], the Provider Self-Assessment Tool, and the Service User Screen Assessment. Exercises focusing on strength and balance include heel raises, toe raises, sit-to-stand, heel-toe stand, one-leg stand, heel-toe walking. Written instructions and images are provided for each of these exercises along with the option to watch video demonstrations for each through the app.

Platform: Android, iOS and windows

Cost: Free

Language: English

Evidence Evaluation: The strength and balance exercises demonstrated through the app have also been included in interventions for which there is an evidence base of effectiveness, such as Otago and FaME. It is unclear if StopFalls was directly based on an existing evidence-based falls prevention programme. There is no evidence regarding the effectiveness of the app in improving strength and balance or preventing falls. Based on two independent ratings there are between 3 and 4 BCTs embedded in the app, including shaping knowledge, natural consequences, comparison of behaviour, and comparison and outcomes. The overall MARS score was 2.8 (acceptable). The highest scoring domain was Functionality (3.9), followed by Information Quality (3.4), Engagement (2.5), and Aesthetics (1.8).

Links: <https://www.hcpastopfalls.info/>

<https://play.google.com/store/apps/details?id=com.hcpastopfalls>

<https://apps.apple.com/us/app/hcpa-stopfalls/id1461092126#?platform=iphone>

<https://www.microsoft.com/en-gb/p/hcpa-stopfalls/9pllc67vfjfb?activetab=pivot:overviewtab>

1. *Wysefit – Wysefit Inc.*

Intention/Aim: The app aims to provide workout classes that older adults can participate in independently in their own homes. The classes aim to help older adults build strength and muscle, lose weight, improve joints health, flexibility and range of motion, gain better balance and functional movement, prevent back pain, and improve mood and cognitive function.

Target Population: Older adults aged over 50

Description: Before beginning any exercises, the user selects goals from a predefined list, which allows the app to build a personalised 21-day or 28-day fitness plan. Both strength and balance are included in the goal list. The fitness plan presents a list of relevant exercise videos to be performed each day. Classes start at beginner level and increase in challenge as training progresses. Some classes require an exercise ball, a chair, or weights and resistance bands.

Platform: iOS

Cost: USD$8.99 per month.

Evidence Evaluation: It is unclear if the specific exercise programme relating to strength and balance is based on an evidence-based intervention. Based on two independent ratings there are between 0 and 3 BCTs embedded in the app, including goals and planning, shaping knowledge, and comparison of behaviour which may facilitate behaviour change and promote adherence. There is no evidence available regarding the effectiveness of the app. The overall MARS score was 3.8 out of 5 (good). Functionality was the highest scoring domain (4.3), followed by Aesthetics (4.2), Engagement (3.5), and Information Quality (3.4).

Links:

<https://www.wysefit.com/>

<https://apps.apple.com/us/app/wysefit-workout-fitness/id1479230988>

*Apps in development*

The following app is still being developed and tested and is not currently available to the public. As such, information on cost is unavailable and information on intended use and platform is subject to change.

1. *StandingTall - NeuRA*

Intention/Aim: StandingTall is a balance training program designed specifically for use by older people. It aims to improving balance and consequently reduce falls and fall risk.

Target Population: Older adults

Description: The StandingTall app was designed for use on a tablet device. The exercises are individually tailored in order to improve balance and reduce fall risk in older people. Users undertake a balance assessment prior to beginning the exercises for the first time. Exercises include high knee marching, heel raises (near tandem), tandem stand, tapping forwards, tapping side, step and bend. In addition to balance exercises, the app also incorporates elements of brain training. The app also employs behavioural change techniques as a means of enhancing uptake and adherence. The app is intended for use by older people independently in their own homes.

Platform: Originally iOS (iPad only), but next iteration will be available on iOS, Android and on a web-based platform.

Language: English

Evidence Evaluation: Many of the exercises featured in the app are also included in interventions for which there is an evidence base of effectiveness, such as Otago and FaME. It is not clear if the StandingTall exercise programme is directly based on one of these interventions, or another evidence-based intervention. The specific exercise programme presented is of unknown effectiveness. An RCT has been completed but results are not yet published [8]. The RCT examines the effectiveness of Standing Tall in community-dwelling older adults aged 70+. An ongoing international implementation study is testing the use of Standing Tall in clinical practice. Based in two independent ratings there are between 8 and 9 BCTs embedded in the app to encourage behaviour change and adherence, including goals and planning, feedback and monitoring, shaping knowledge, comparison of behaviour, associations, and repetition and substitution. The overall MARS score was 3.8 indicating good overall quality. The highest scoring domain was Aesthetics (4.2) followed by Functionality (4.0), Information Quality (3.8), and Engagement (3.2).

Links: Clinical trial 2015 protocol in BMJ: <https://bmjopen.bmj.com/content/5/10/e009173?int_source=trendmd&int_medium=trendmd&int_campaign=trendmd>

For more information see - <https://www.standingtall.org.au/>

<https://apps.apple.com/au/app/standingtall/id1468991305>

### Websites

1. *Age UK, UK - ageuk.org.uk*

Intention/Aim: AgeUK aims to provide companionship, advice, and support to older adults in the UK.

Target Population: Older adults

Description: The website contains a link to an exercise booklet (available as PDF) that describes and demonstrates falls prevention strength and balance exercises. The booklet contains descriptions, accompanied by images, of a number of warm up exercises including chair march, arm swings, shoulder circles, ankle loosener, spine twists, chest stretch, back of thigh stretch and calf stretch. Strength exercises described and demonstrated using images include wrist strengthener, sit to stand, upper back strengthener, thigh strengthener, and wall press-up. Balance exercises described and demonstrated using images include side steps, heel raises, toe raises, marching, and leg swings.

Platform: Website

Cost: Free

Language: English

Evidence Evaluation: The exercises described and demonstrated in the booklet have also been included in interventions for which there is evidence of effectiveness (e.g. FaME and Otago). There is no evidence to support the effectiveness of this website in improving strength and balance or preventing falls. Based on two independent ratings there were three BCTs relating to natural consequences, shaping knowledge, and comparison of behaviour. Overall website quality was scored as excellent (87%).

Links:

Main website: <https://www.ageuk.org.uk/>

Strength and balance PDF: <https://www.ageuk.org.uk/Documents/EN-GB/strength_and_balance_training_PDF.pdf?dtrk=true>.

1. *Better Health While Aging, USA - betterhealthwhileaging.net*

Intention/Aim: Better Health While Aging (formerly named Geriatrics For Caregivers) is an information website for older adults and caregivers.

Target Population: Older adults and caregivers

Description: The website provides videos demonstrating exercises used as part of the Otago Exercise Programme for Fall Prevention. Videos include assessment exercises (Timed Up and Go, 30-second chair stand, 4-stage balance test,) warm up exercises (head movements, neck movements, back extensions, trunk movements, ankle extensions), strength exercises (front knee strengthening, back knee strengthening, side hip strengthening, calf raises, toe raises), and balance exercises (knee bends, backwards walk, walking and turning around, sideways walking, heel-toe stand, heel-toe walking, one-leg stand, heel walking, toe walking, heel-toe walking backwards, sit to stand, stair walking).

Platform: Website; Videos are also available on YouTube

Cost: Free

Language: English. Some videos available in Spanish.

Evidence Evaluation: The exercise programme demonstrated on this website is based on the Otago Exercise Programme, for which there is a strong evidence-base of effectiveness. There is no evidence regarding the effectiveness of the website itself in improving strength and balance or preventing falls. Based on two independent ratings there were four BCTs relating to shaping knowledge, natural consequences, comparison of behaviour, repetition and substitution, and comparison and outcomes. Overall website quality was scored as good (66%).

Links: <https://betterhealthwhileaging.net/otago-exercises-fall-prevention-videos/>

1. *Buffalo Rehab, USA – buffalorehab.com*

Intention/Aim: Buffalo Rehab Group are a physical therapy business based in the United States. Their website features a blog discussing a range of issues relevant to physical therapy.

Target Population: The general population, with some content directed at older adults.

Description: One blog post on the website demonstrates two balance exercises for older adults, in the form of an images, in order to prevent falls and fall related injuries. Demonstrated exercises are: narrowing the base of support and tandem stand.

Platform: Website

Cost: Free

Language: English

Evidence Evaluation: The two exercises demonstrated on this website have also been included in interventions for which there is a strong evidence base of effectiveness, such as FaME and Otago. There is no evidence regarding the effectiveness of this website in improving strength and balance or preventing falls. Based on two independent ratings there were between four and five BCTs relating to shaping knowledge, natural consequences, comparison of behaviour, repetition and substitution, and comparison and outcomes. Overall website quality was scored as good (68%).

Links: <https://buffalorehab.com/blog/tips-to-improve-your-balance/#targetText=The%20next%20exe rcise%20you%20can,posture%20and%20maintain%20your%20balance.&targetText=If%20you%20are%20able%20to,can%20try%20single%20limb%20stance.>

1. *Caregiver Stress, USA - caregiverstress.com*

Intention/Aim: The Caregiver Stress website is run by Home Instead Senior Care, a franchised in-home care agency. Website resources are generally directed at caregivers and cover self-care and senior care. The website aims to help carers manage, minimize, or prevent stress.

Target Population: Carers of older adults

Description: The website provides descriptions and demonstrations of six exercises that can be performed at home to reduce senior fall risk. The exercises are described in text and contain links to videos demonstrating each exercise that were developed in conjunction with the University of Nebraska Medical Centre. Exercises include side leg raises, weight shifts, heel-toe stands, one-leg stand, heel-toe walking, and walking on toes.

Platform: Website

Cost: Free

Language: English

Evidence Evaluation: The exercises described and demonstrated on the website have also been included in interventions for which there is evidence of effectiveness (e.g. FaME and Otago). There is no evidence to support the effectiveness of this website in improving strength and balance or preventing falls. Based on two independent ratings there were three BCTs relating to shaping knowledge, natural consequences, and comparison of behaviour. Overall website quality was scored as good (72%).

Links: <https://www.caregiverstress.com/geriatric-professional-resources/share-clients/6-easy-at-home-exercises-to-reduce-senior-fall-risk/>

1. *Caring Senior Service, USA - caringseniorservice.com*

Intention/Aim: Caring Senior Service is a caregiver franchise that helps seniors in their own homes. The company aims to help seniors remain healthy and happy at home.

Target Population: Older adults or caregivers of older adults.

Description: The website has a blog section that contains information on the benefits of exercise in falls prevention as well as links to videos of an Otago exercise class and seated stretch demonstrations. The site also includes a link to an infographic on balance training exercises, including balance with ball toss, contralateral and ipsilateral marching, squats, and step-ups, accompanied by images and written descriptions.

Platform: Website; Video links are played through YouTube.

Cost: Free

Language: English

Evidence Evaluation: The website provides a link to a demonstration of the evidence-based Otago exercise programme. Some of the balance exercises outlined in the infographic have also been included in interventions for which there is evidence of effectiveness, such as FaME and Otago. There is no evidence regarding the effectiveness of this website in improving strength and balance or preventing falls. Based on two independent ratings there were between two and three BCTs relating to shaping knowledge, natural consequences, and comparison of behaviour. Overall website quality was scored as fair (50%).

Links: <https://www.caringseniorservice.com/blog/great-fall-prevention-exercises-for-seniors>

Otago exercise class video: <https://www.youtube.com/watch?v=z6JoaJgofT8>

Seated stretches: <https://www.youtube.com/watch?v=CJrpkOj0z4M>

Infographic on balance training: <https://www.caringseniorservice.com/blog/infographic-balance-exercises-for-seniors>

1. *Closing the Gap Healthcare, Canada - closingthegap.ca*

Intention/Aim: Closing the Gap healthcare provides a range of services for people of all ages, particularly in relation to complex care. Personalised care is tailored to individual needs.

Target Population: The general population, including older adults.

Description: In addition to information on their services, the website provides a blog that contains information on various health issues. One blog article outlines 10 fall prevention exercises that older people can perform while seated. The exercises, described in text and using images, include stretches, arm raises, hand squeezes, tummy twists, side bends, and knee extensions.

Platform: Website

Cost: Free

Language: English

Evidence Evaluation: There is little robust evidence to suggest that chair-based exercises are effective with regard to falls prevention [9]. There is no evidence to support the effectiveness of this website in improving strength and balance or preventing falls. Based on two independent ratings there were two BCTs relating to shaping knowledge and natural consequences. Overall website quality was scored as fair (58%).

Links: <https://www.closingthegap.ca/blog/10-fall-prevention-exercises-seniors-can-do-while-sitting-in-a-chair/>

1. *Chartered Society of Physiotherapy (CSP, UK - csp.org.uk*

Intention/Aim: The Chartered Society of Physiotherapy (CSP) provides videos and images on their websites that were developed by Agile, the CSP’s professional network for physiotherapists working with older people. The aim is to demonstrate exercises that can help improve balance and build strength to reduce falls risk. Images demonstrating the exercises are also included in the Get Up And Go booklet (PDF available to download on the website).

Target Population: The animation is aimed at the general public (aged 55+). It can also be used by physiotherapists and other health professionals working with older adults.

Description: CSP animated videos and images demonstrate how six exercises, often used by physiotherapists, can strengthen muscles and improve balance. Exercises include heel raises, toe raises, heel toe stand, one leg stand, heel toe walking, and sit-to-stand, often referred to as the “super six”. The videos are available to watch on the website and are also available on YouTube.

Platform: Website; YouTube.

Cost: Free

Language: English

Evidence Evaluation: The exercise videos were developed with Agile, the CSP’s professional network for physiotherapists working with older people. The exercises presented are used in evidence-based programmes, such as FaME and Otago. There is no evidence for the effectiveness of the website itself in reducing or preventing falls. Based on two independent ratings there were five BCTs relating to shaping knowledge, natural consequences, comparison of behaviour, and comparison and outcomes. Overall website quality was scored as excellent (86%).

Links:

Website: [www.csp.org.uk](http://www.csp.org.uk)

Videos: <https://www.csp.org.uk/news/2017-09-27-csp-launches-video-demonstrate-six-simple-exercises-stop-falls>

PDF booklet: <https://www.csp.org.uk/publications/get-and-go-guide-staying-steady-english-version>. Also available on Centre for Ageing Better website: <https://www.ageing-better.org.uk/strength-balance-resources>

1. *Daily Caring, USA – dailycaring.com*

Intention/Aim: Daily Caring is an American website aimed at caregivers for older adults. The site, which is associated with the Institute on Aging, mainly provides support and information on various issues affecting caregivers. The website features a video demonstrating 10 simple fall prevention exercises, focusing on balance, that older adults can do at home.

Target Population: Older adults or caregivers of older adults

Description: Two physical therapists demonstrate 10 balance exercises older adults can do at home. Exercises include sit-to-stand; one-hand sit-to-stand; hands-free sit-to stand; leg raises; marching; hip extension; heel-toe standing; one-leg stand; heel-toe walking.

Platform: Website; Videos are also available on YouTube

Cost: Free

Language: English

Evidence Evaluation: The exercises demonstrated on this website have also been included in interventions for which there is a strong evidence base of effectiveness, such as FaME and Otago. There is no evidence regarding the effectiveness of this website in improving strength and balance or preventing falls. Based on two independent ratings there were four BCTs relating to shaping knowledge, natural consequences, comparison of behaviour, repetition and substitution, and comparison and outcomes. Overall website quality was scored as good (71%).

Links: <https://dailycaring.com/10-simple-fall-prevention-exercises-seniors-can-do-at-home-video/>

1. *Elder Gym, USA - eldergym.com*

Intention/Aim: The aim of Elder Gym is to help seniors remain independent and strong by providing useful resources to help people stay fit and active as they age.

Target Population: Older adults

Description: The website provides information on falls and fall prevention as well as detailed descriptions and demonstrations (images and videos) of 12 balance exercises that can be performed at home. The exercises include single leg stand, eye tracking, clock reach, staggered stance, single leg with arm, balancing wand, knee marching, body circles, heel to toe, grapevine, stepping, and dynamic walking.

Platform: Website

Cost: Free

Language: English

Evidence Evaluation: Many of the exercises described and demonstrated on the website have also been included in interventions for which there is evidence of effectiveness (e.g. FaME and Otago). There is no evidence to support the effectiveness of this website in improving strength and balance or preventing falls. Based on two independent ratings there were five BCTs relating to shaping knowledge, natural consequences, comparison of behaviour, repetition and substitution, and comparison and outcomes. Overall website quality was scored as fair (60%).

Links: <https://eldergym.com/elderly-balance/>

1. *Exercise Right, Australia - exerciseright.com.au*

Intention/Aim: Exercise Right is a public awareness campaign created by Exercise & Sports Science Australia. The website aims to motivate those who don’t exercise to move more, while also demonstrating to people the importance of seeking the right exercise expert for their requirements.

Target Population: The general population, including older adults

Description: The website has an “Active Ageing” category where information on falls prevention exercises is provided. Under this category, three exercises are described that are designed to be performed at home. These include heel toe balance, sideways walking, and marching on the spot. The exercises are described in text and accompanying images. In addition, the website has a ‘Home Workout’ category, where two falls prevention videos are provided. The videos are led by accredited exercise physiologists and the demonstrated exercises include sit to stand; calf raises; semi-tandem stand; tandem stand; one leg stand; knee extensions; shoulder rolls; arm raises; ankle rolls; marching in place; heel kicks; side leg raises; forwards and backwards heel toe walking.

Platform: Website

Cost: Free

Language: English

Evidence Evaluation: The exercises demonstrated on the website have also been included in interventions for which there is evidence of effectiveness (e.g. FaME and Otago). There is no evidence to support the effectiveness of this website in improving strength and balance or preventing falls. Based on two independent ratings there were two BCTs relating to shaping knowledge, natural consequences, and comparison of behaviour. Overall website quality was scored as excellent (81%).

Links:

Fall prevention exercises: <https://exerciseright.com.au/keeping-simple-falls-prevention-exercises-home/>

Falls prevention videos: <https://exerciseright.com.au/home-workouts-active-ageing/>

1. *Scottish Centre for Telehealth and Telecare (SCTT), UK - fallsassistant.org.uk*

Intention/Aim: The aim of Falls Assistant is to help prevent falls, keep people steady on their feet and stay as independent as possible.

Target Population: Older people, their carers and family members.

Description: The website enables the user to assess their falls risk and provides tailored advice and information on falls and fall prevention in text format, as well as games to help identify and learn about fall hazards in and around the home. The website includes descriptions and video demonstrations of strength and balance exercises tailored to ability level. Exercises include heel raises, toe raises, heel toe stand, heel toe walking, flamingo swings, toe walk, heel walk, tandem walk, side stepping, sit to stand, knee lifts/marching, and knee bends.

Platform: Website

Cost: Free

Language: English

Evidence Evaluation: This resource has been developed by NHS 24’s Scottish Centre for Telehealth and Telecare (SCTT) as part of the European SmartCare programme. The strength and balance exercises demonstrated on the website have also been included in interventions for which there is an evidence base of effectiveness, such as FaME and Otago. However, there is currently no evidence regarding the effectiveness of this website in improving strength and balance or preventing falls. Based on two independent ratings there were seven BCTs relating to goals and planning, shaping knowledge, natural consequences, comparison of behaviour, repetition and substitution, and comparison and outcomes. Overall website quality was scored as excellent (79%).

Links: <https://fallsassistant.org.uk/>

1. *Go4Life, The National Institute on Aging, USA – go4life.nia.nih.gov*

Intention/Aim: Go4Life, an exercise and physical activity campaign from the National Institute on Aging in the United States, is designed to help older adults incorporate exercise and physical activity into their daily lives.

Target Population: Older adults (aged 50+)

Description: The website includes images and videos promoting four types of activities that can help older adults stay independent – endurance, strength, balance, and flexibility. Strength exercises target both upper and lower body and include chair dip, wall push-up, toe stand, chair stand, and leg raises. Balance exercises include one foot balance (with chair support), heel-to-toe walk exercise, balance walk exercise. Tai chi is also promoted in a demonstration video.

Platform: Website; Videos are also available on YouTube

Cost: Free

Language: English

Evidence Evaluation: Most of the strength and balance exercises demonstrated on the website have also been included in interventions for which there is an evidence base of effectiveness (e.g. FaME and Otago). There is no evidence regarding the effectiveness of this website in improving strength and balance or preventing falls. Based on two independent ratings there were between four and five BCTs relating to shaping knowledge, natural consequences, comparison of behaviour, and comparison and outcomes. Overall website quality was scored as good, bordering on excellent (75%).

Links: <https://go4life.nia.nih.gov/4-types-of-exercise/>

<https://www.youtube.com/watch?v=P_GPWLlQVMw>

At the time of review the website was transitioning its exercise and physical activity information and resources to the NIA Exercise and Physical Activity website. Once complete, the Go4Life website will automatically redirect to this site: <https://www.nia.nih.gov/health/exercise-physical-activity>

1. *Health Hub, Singapore - healthhub.sg*

Intention/Aim: Health Hub is a website for Singaporeans to access a wide range of health information and e-services. Health Hub is an initiative of the Ministry of Health and the Health Promotion Board in Singapore.

Target Population: The general population, including older adults

Description: The website provides descriptions and demonstrations (both images and videos) of 7 simple strength and balance exercises suitable for older adults. The exercises include stationary march with arm swing, sit to stand, standing hip extension, side leg raise, single leg stand, triceps stretch, and standing quadriceps stretch.

Platform: Website

Cost: Free

Language: Malay, Chinese, Tamil, English

Evidence Evaluation: The exercises described and demonstrated on the website have also been included in interventions for which there is evidence of effectiveness (e.g. FaME and Otago). There is no evidence to support the effectiveness of this website in improving strength and balance or preventing falls. Based on two independent ratings there were four BCTs relating to shaping knowledge, natural consequences, comparison of behaviour, and comparison and outcomes. Overall website quality was scored as good (73%).

Links: <https://www.healthhub.sg/programmes/71/healthy-ageing-exercise>

<https://www.healthhub.sg/programmes/83/healthy-ageing-exercise-step-by-step-guide>

1. *Health Line, USA - healthline.com*

Intention/Aim: The aim of the website is to make health and wellness information accessible, understandable, and actionable so that readers can make the best decisions about their health. All content is reviewed by medical professionals to ensure accuracy.

Target Population: The general population, including older adults.

Description: The website describes and demonstrates three balance exercises to prevent falls that are suitable for seniors. Exercises are described in text and in images and include tightrope walking (tandem walking), rock the boat and flamingo stand (one-leg stand).

Platform: Website

Cost: Free

Language: English

Evidence Evaluation: Most of the exercises described and demonstrated on the website have also been included in interventions for which there is evidence of effectiveness (e.g. FaME and Otago). There is no evidence to support the effectiveness of this website in improving strength and balance or preventing falls. Based on two independent ratings there were four BCTs relating to shaping knowledge, natural consequences, comparison of behaviour, and repetition and substitution. Overall website quality was scored as excellent (78%).

Links: <https://www.healthline.com/health/exercises-for-balance#exercise-for-seniors>

1. *Health Link BC, Canada - healthlinkbc.ca*

Intention/Aim: This website provides medically approved information on health issues and symptoms. It includes interactive health tools and tips for maintaining a healthy lifestyle.

Target Population: General population, including older adults

Description: The site provides information on strength and balance exercises to prevent falls. The guidance begins with some warm up exercises, including shoulder rolls, calf stretches, and marching in place, with links to images demonstrating the exercises. Descriptions of exercises to improve strength include leg bends, knee bends (link to images demonstrating the exercise), heel raises (link to images demonstrating the exercise), leg lifts, and sit to stand. Only written descriptions are provided for exercises to improve balance. The exercises include one-leg balance, toe-heel balance, hip circles, and heel toe walking.

Platform: Website

Cost: Free

Language: English, Arabic, Chinese, Farsi, French, Korean, Punjabi, Spanish, Vietnamese.

Evidence Evaluation: The exercises described and demonstrated on the website have also been included in interventions for which there is evidence of effectiveness (e.g. FaME and Otago). There is no evidence to support the effectiveness of this website in improving strength and balance or preventing falls. Based on two independent ratings there were between two and three BCTs relating to shaping knowledge, natural consequences, and comparison of behaviour. Overall website quality was scored as good (73%).

Links: <https://www.healthlinkbc.ca/health-topics/av2500>

1. *Johns Hopkins Medicine, USA - hopkinsmedicine.org*

Intention/Aim: Johns Hopkins Medicine website aims to set standards of excellence in medical information, research and clinical care. The Wellness and Prevention section of the website provides general information on prevention in relation to a range of health issues, including falls in older adults.

Target Population: The general population, with some content directed at older adults.

Description: The website includes four videos demonstrating sit-to-stand exercise, and a series of balance exercises, including standing with feet apart, standing with feet together, standing on one foot with eyes open, and standing on one foot with eyes closed. The videos are led by a physical therapist from the John Hopkins Rehabilitation Network. The exercises are intended for those who are able to stand on their own without support and are at low risk of falling.

Platform: Website

Cost: Free

Language: English

Evidence Evaluation: The strength and balance exercises demonstrated on the website have also been included in interventions for which there is an evidence base of effectiveness, such as FaME and Otago. There is currently no evidence regarding the effectiveness of this website in improving strength and balance or preventing falls. Based on two independent ratings there were four BCTs relating to shaping knowledge, natural consequences, comparison of behaviour, and comparison and outcomes. Overall website quality was scored as good (68%).

Links: <https://www.hopkinsmedicine.org/health/wellness-and-prevention/fall-prevention-exercises>

1. *Mayo Clinic, USA – mayoclinic.org*

Intention/Aim: The Mayo Clinic is non-profit medical organisation in the United States focused on clinical practice, education and research. Their website contains broad information on how to maintain a healthy lifestyle.

Target Population: The general population, with some content directed at older adults.

Description: The website features images that demonstrate strength and balance exercises for older adults in order to prevent falls and maintain independence. The images illustrate three types of exercises for strength and balance: Weight shifts, single-leg balance and bicep curls with dumbbell.

Platform: Website

Cost: Free

Language: English

Evidence Evaluation: Most of the exercises demonstrated on this website have also been included in interventions for which there is evidence of effectiveness (e.g. FaME and Otago). There is no evidence regarding the effectiveness of this website in improving strength and balance or preventing falls. Based on two independent ratings there were between one and three BCTs relating to shaping knowledge, comparison of behaviour, and comparison and outcomes. Overall website quality was scored as excellent (85%).

Links: <https://www.mayoclinic.org/healthy-lifestyle/fitness/multimedia/balance-exercises/sls-20076853?s=1>

1. *Melio Guide Physical Therapy, USA - melioguide.com*

Intention/Aim: Melio Guide Physical Therapy provides physical therapy services and programmes for the prevention and treatment of osteoporosis. The website aims to provide general information and resources on exercises to help build stronger bones. They also provide guidance on strategies for fall prevention.

Target Population: The general public, including older adults

Description: The website contains a section that provides links to video demonstrations of balance exercises for seniors and a fall prevention checklist. The exercises demonstrated include dynamic standing, single leg stand, single leg reach, balance ball exercises, and tai chi.

Platform: Website

Cost: Free

Language: English

Evidence Evaluation: Most of the exercises described and demonstrated on the website have also been included in interventions for which there is evidence of effectiveness (e.g. FaME and Otago). There is no evidence to support the effectiveness of this website in improving strength and balance or preventing falls. Based on two independent ratings there were six BCTs relating to shaping knowledge, natural consequences, comparison of behaviour, repetition and substitution, and comparison and outcomes. Overall website quality was scored as good (64%).

Links: <https://melioguide.com/health-guides/balance-exercises-for-seniors/>

1. *National Health Service (NHS), UK – nhs.uk/live-well*

Intention/Aim: The Live Well section of the NHS website aims to provide general advice and tools to help people make good choices surrounding their health and wellbeing. Exercise advice and demonstrations are provided for all age groups, including over 65s.

Target Population: The general population, with some content directed at older adults.

Description: The website includes graphics that demonstrate strength and balance exercises step-by-step. Strength exercises are sit-to-stand, mini-squats, calf raises, sideways leg lift, leg extension; wall press up; biceps curl. Balance exercises are heel-to-toe walk, sideways walking, grapevine, and one-leg stand; step-up. The routines can be downloaded as PDFs. The website also provides a 5-week exercise plan designed to improve strength and flexibility, delivered via podcasts. The podcasts were developed by ‘YMCA fit’ in response to the Department of Health physical activity guidelines, which state that adults aged 19-64 should engage in muscle strengthening activities at least twice a week. As such, these exercises are likely only suitable for older adults aged under 65 years.

Platform: Website

Cost: Free

Language: English

Evidence Evaluation: The strength and balance exercises demonstrated on the website have also been included in interventions for which there is an evidence base of effectiveness, such as FaME and Otago. There is currently no evidence regarding the effectiveness of this website in improving strength and balance or preventing falls. Based on two independent ratings there were between two and four BCTs relating to goals and planning, shaping knowledge, comparison of behaviour, and comparison and outcomes. Overall website quality was scored as excellent (81%).

Links: Strength: <https://www.nhs.uk/live-well/exercise/strength-exercises/>

Balance: <https://www.nhs.uk/live-well/exercise/balance-exercises/>

Strength and Flexibility: <https://www.nhs.uk/live-well/exercise/strength-and-flex-exercise-plan/>

1. *National Health Service (NHS) Inform, UK – nhsinform.scot*

Intention/Aim: The NHS Inform website includes a section on healthy living to provide information and advice on ways people can look after their physical and mental health. Specific information is provided on falls prevention in older adults, including the importance of exercise.

Target Population: The general population, with some content directed at older adults.

Description: The website includes five demonstrational videos of simple exercises people can perform in their own homes:

1. Introduction to exercise.

2. Testing your balance: this video demonstrates some simple balance exercises using a chair in order to establish which of the exercise levels you should begin with.

3. Level 1 exercises: easy marching; trunk movements; front knee strengthener; side hip strengthener; calf or heel raises; toe raises; sit to stand.

4. Level 2 exercises: easy marching; ankle movements; back knee strengthening; knee bends; heel-toe standing; heel-toe walking; sideways walking; sit to stand.

5. Level 3 exercises: easy marching; ankle movements; toe raises; toe walking; heel walking; sit to stand.

Platform: Website

Cost: Free

Language: English

Evidence Evaluation: The strength and balance exercises demonstrated on the website have also been included in interventions for which there is an evidence base of effectiveness, such as FaME and Otago. There is currently no evidence regarding the effectiveness of this website in improving strength and balance or preventing falls. Based on two independent ratings there were seven BCTs relating to shaping knowledge, natural consequences, comparison of behaviour, repetition and substitution, and comparison and outcomes. Overall website quality was scored as excellent (83%).

Links: <https://www.nhsinform.scot/healthy-living/preventing-falls/keeping-well/strength-and-balance-exercises>

1. *Paths for All, UK - pathsforall.org.uk*

Intention/Aim: Paths for All is a Scottish charity that champions daily walking in order to improve the general health of the population.

Target Population: The general population, including older adults

Description: Paths for All developed a “Walk Your Way to Strength and Balance” programme with expert input from Glasgow Caledonian University. The programme consists of strength and balance exercises to reduce risk of falls and a brochure describing and demonstrating the exercises using images is available to download on their website. Videos of the exercises are also available through YouTube. The exercises include front knee strengthener, back knee strengthener, knee bends, sit to stand, calf raises with support, toe raises with support, side hip strengthener, sideways walking, toe walking with no support, heel walking with no support.

Platform: Website; videos available through YouTube

Cost: Free

Language: English

Evidence Evaluation: The exercises described and demonstrated on the website have also been included in interventions for which there is evidence of effectiveness (e.g. FaME and Otago). There is no evidence to support the effectiveness of this website in improving strength and balance or preventing falls. Based on two independent ratings there were four BCTs relating to shaping knowledge, natural consequences, comparison of behaviour, repetition and substitution, and comparison and outcomes. Overall website quality was scored as good (72%).

Links: <https://www.pathsforall.org.uk/strength-and-balance-exercises>

Videos: <https://www.youtube.com/playlist?list=PLZ3bXGWNVY4LDwJGvyIFh61OpyMqCMEc8>

1. *Staying on your Feet: Taking Steps to Prevent Falls, Canada - preventfalls.ca*

Intention/Aim: This website is run by the Winnipeg Regional Health Authority in Canada. The website aims to provide education about falls and falls prevention in both older adults and children.

Target Population: General population, with the main focus being placed on older adults

Description: The website contains written descriptions, accompanied by picture demonstrations, for balance exercises that can be conducted in the home. The exercises include tandem stance, side leg raises, toe stands, knee lifts, sit-to-stand.

Platform: Website

Cost: Free

Language: English and French

Evidence Evaluation: The balance exercises described on this website have also been included in interventions for which there is evidence of effectiveness, such as FaME and Otago. There is no evidence regarding the effectiveness of this website in improving strength and balance or preventing falls. Based on two independent ratings there were between one and three BCTs relating to shaping knowledge, natural consequences, and comparison of behaviour. Overall website quality was scored as good (69%).

Links: <https://preventfalls.ca/older-adults/exercise/home-exercise/>

1. *ProFouND Falls Prevention Network**, Europe - profound.eu.com*

Intention/Aim: ProFouND is an EU funded Thematic Network focusing on falls prevention. The aim of ProFouND is to influence policy and increase awareness of falls and falls prevention programmes.

Target Population: Older adults and organisations working with older people.

Description: The ProFouND website contains numerous videos ranging from exercise demonstrations to general information on falls prevention. Five of the exercise demonstration videos were developed by FARSEEING, a collaborative European Commission funded research project with 10 partners across 5 EU countries. FARSEEING aims to promote healthy ageing and independent living for older adults, with the specific aim of preventing falls. The videos demonstrate the following exercises: heel raises, sit to stand, seated knee strengthener, shoulder mobility and side hip strengthener. There is also an Otago demonstration video provided on the ProFouND website, and strength and balance exercise demonstration videos in Italian, French and German languages. The website also includes numerous videos of seminars, lectures, and general information on falls prevention.

Platform: Website

Cost: Free

Language: English; Spanish. Exercise videos in a number of European languages, including: English, Italian, French, German.

Evidence Evaluation: The website presents a video demonstrating exercises from the Otago programme, an intervention for which there is strong evidence of effectiveness. Exercises presented in strength and balance videos are used in evidence-based programmes, such as FaME and Otago. There is no evidence for the effectiveness of the website itself in reducing or preventing falls. Based on two independent ratings there were five BCTs relating to shaping knowledge, natural consequences, comparison of behaviour, repetition and substitution, and comparison and outcomes. Overall website quality was scored as good (64%).

Links:

Exercise and educational videos: <http://profound.eu.com/category/videos/page/2/>

Further information on FARSEING: <http://farseeingresearch.eu/>

1. *Unity Point, USA - unitypoint.org*

Intention/Aim: Unity Point is an integrated healthcare service in the USA providing health care across three states. The aim of the website is to outline the services available to patients as well as general health information.

Target Population: The general public, including older adults.

Description: The website describes and demonstrates a series of balance exercises to prevent falls using animated videos. Exercises include glute bridge; hip abduction, flexion and extension; sit to stand; scapular rows; tandem stand; and one leg stand.

Platform: Website

Cost: Free

Language: English

Evidence Evaluation: Most of the exercises described and demonstrated on the website have also been included in interventions for which there is evidence of effectiveness (e.g. FaME and Otago). There is no evidence to support the effectiveness of this website in improving strength and balance or preventing falls. Based on two independent ratings there were three BCTs relating to shaping knowledge, natural consequences, comparison of behaviour, repetition and substitution, and comparison and outcomes. Overall website quality was scored as fair (59%).

Links: <https://www.unitypoint.org/livewell/article.aspx?id=91b1a159-d94a-4ba2-b64e-81236a3d538a>

References

1. Dewick P, Stanmore EK. Applying Game Thinking to Slips, Trips and Falls Prevention. In: Cudd P, de Witte L (eds.). *Harnessing the Power of Technology to Improve Lives*. Amsterdam: IOS Press, 2017, 606–13.

2. Stanmore EK, Dewick P. The development of a gamified digital health application to improve knowledge and influence behaviour to prevent falls, increase exercise levels and improve bone health in older people. Manchester, United Kingdom, 2018.

3. Stanmore EK, Fisher F, Dewick P *et al.* Feasibility, usability and acceptability of a gamified strength and balance training and healthy literacy platform (Keep-On-Keep-Up) for older adults living in the community. in preparation.

4. Yardley L, Beyer N, Hauer K *et al.* Development and initial validation of the Falls Efficacy Scale-International (FES-I). *Age Ageing* 2005;**34**:614–9.

5. Le Berre M, Apap D, Babcock J *et al.* The Psychometric Properties of a Modified Sit-to-Stand Test With Use of the Upper Extremities in Institutionalized Older Adults. *Percept Mot Skills* 2016;**123**:138–52.

6. CDC. *STEADI - Older Adult Fall Prevention*. Centers for Disease Control and Prevention, 2019.

7. Nandy S, Parsons S, Cryer C *et al.* Development and preliminary examination of the predictive validity of the Falls Risk Assessment Tool (FRAT) for use in primary care. *J Public Health* 2004;**26**:138–43.

8. Delbaere K, Valenzuela T, Woodbury A *et al.* Evaluating the effectiveness of a home-based exercise programme delivered through a tablet computer for preventing falls in older community-dwelling people over 2 years: study protocol for the Standing Tall randomised controlled trial. *BMJ Open* 2015;**5**, DOI: 10.1136/bmjopen-2015-009173.

9. Anthony K, Robinson K, Logan P *et al.* Chair-Based Exercises for Frail Older People: A Systematic Review. *BioMed Res Int* 2013;**2013**:e309506.
